# The patient and clinician experience of informed consent for surgery: a systematic review of the qualitative evidence

**DOI:** 10.1186/s12910-020-00501-6

**Published:** 2020-07-11

**Authors:** L. J. Convie, E. Carson, D. McCusker, R. S. McCain, N. McKinley, W. J. Campbell, S. J. Kirk, M. Clarke

**Affiliations:** 1 Department of General Surgery, Ulster Hospital Dundonald, Belfast, UK; 2 Centre for Public Health, Queen’s University Belfast, Belfast, UK

**Keywords:** Informed consent, Surgery, Adults, Qualitative synthesis

## Abstract

**Background:**

Informed consent is an integral component of good medical practice. Many researchers have investigated measures to improve the quality of informed consent, but it is not clear which techniques work best and why. To address this problem, we propose developing a core outcome set (COS) to evaluate interventions designed to improve the consent process for surgery in adult patients with capacity. Part of this process involves reviewing existing research that has reported what is important to patients and doctors in the informed consent process.

**Methods:**

This qualitative synthesis comprises four phases: identification of published papers and determining their relevance; appraisal of the quality of the papers; identification and summary of the key findings from each paper while determining the definitiveness of each finding against the primary data; comparison of key themes between papers such that findings are linked across studies.

**Results:**

Searches of bibliographic databases returned 11,073 titles. Of these, 16 studies met the inclusion criteria. Studies were published between 1996 and 2016 and included a total of 367 patients and 74 health care providers. Thirteen studies collected data using in-depth interviews and constant comparison was the most common means of qualitative analysis. A total of 94 findings were extracted from the primary papers and divided into 17 categories and ultimately 6 synthesised findings related to: patient characteristics, knowledge, communication, the model patient, trust and decision making.

**Conclusions:**

This qualitative meta-aggregation is the first to examine the issue of informed consent for surgery. It has revealed several outcomes deemed important to capture by patients and clinicians when evaluating the quality of a consent process. Some of these outcomes have not been examined previously in research comparing methods for informed consent. This review is an important step in the development of a COS to evaluate interventions designed to improve the consent process for surgery.

**Registration:**

The study protocol was registered on the international prospective register for systematic reviews (PROSPERO ID: CRD42017077101).

## Background

Informed consent is integral to good medical practice [1]. Prior to any examination or treatment, clinicians should discuss with patients the potential harms, benefits and alternatives of the proposed care [2]. Shortcomings in this process can lead to a breakdown in the clinician-patient relationship and occasionally litigation. A series of high profile court proceedings have redefined how the consent process is conducted [3–5]. The courts now expect doctors to advise their patients of all risks that an individual patient would determine to be of ‘material’ importance to him or her, regardless of how unlikely that risk might be [4]. This poses an almost impossible task for healthcare providers within the confines of the current model of care provision in most health services internationally.

To address this issue, many researchers have investigated how the quality of informed consent might be improved. A Cochrane review demonstrated that interventions designed to improve the quality of informed consent appear to work [6]. However, because no two studies have assessed the effects of their intervention in the same way, it has been impossible to make direct comparisons between studies or to synthesise the data with any meaning. This qualitative synthesis is part of a larger piece of work to determine which factors are ‘core’ in determining the quality of informed consent [7]. It is envisaged that the development of a core outcome set (COS) would encourage standardised reporting of outcomes to address the issues highlighted by the Cochrane review.

Practicing evidence-based medicine is fundamental to good medical practice [1]. It is a process through which clinicians find, critique and use medical research to answer clinical questions [8]. Well conducted systematic reviews of the evidence are viewed as the corner-stone on which evidence-based healthcare is built. Historically, systematic reviews have been designed to answer questions regarding the effectiveness of interventions and therefore have involved the analysis and synthesis of several quantitative studies. However, the value of systematic reviews of qualitative evidence has been increasingly recognised as providing important context for quantitative data as well as answering some healthcare questions in their own right [9, 10].

The debate as to whether qualitative data can or even should be synthesised has largely sided in support of synthesis. However, there is no internationally recognised singular method to combine the findings of qualitative studies, as illustrated by the numerous methodologies available for this purpose. However, groups such as Cochrane and the Joanna Briggs Institute have provided a forum for discussion regarding the development of methodology in this area and offer examples of good practice [10, 11].

The aim of this qualitative synthesis is to determine which domains determine the quality of informed consent for surgery as viewed by patients and clinicians. In evaluating the qualitative literature in this field in a systematic way, we believe we can construct a picture of the factors which are important to both patients and their doctors during the informed consent process. This information may reveal some potential outcome measures that have not yet been reported elsewhere for prioritisation in the development of a COS.

## Review question

What are the experiences, concerns and needs of patients and clinicians during the informed consent process for surgery or other invasive healthcare procedures?

## Methods

This qualitative synthesis comprises four phases: identification of published papers and determining their relevance; appraisal of the quality of the papers; identification and summary of the key findings from each paper while determining the definitiveness of each finding against the primary data; comparison of key themes between papers such that findings are linked across studies [12–14]. The study protocol was registered on the international prospective register for systematic reviews (PROSPERO ID: CRD42017077101) [15].

### Identification of published studies

The search strategy was developed among the authors with the assistance of a senior medical librarian. A systematic search was conducted across Medline (1946 to 13 September 2017), EMBASE (1974 to 2017 Week 37), CINAHL (1982 to 14 September 2017), Cochrane Library (on 14 September 2017), Web of Science (on 12 September 2017) and PsycInfo (1806 to first week September 2017). The full search strategy for each database is available in Additional File 1.

Performing a systematic search for relevant qualitative studies can be fraught with difficulty. While search filters for qualitative studies do exist in some databases, many of these have only been introduced in recent years and the indexing of qualitative studies is often incomplete [16]. With this in mind, search terms to return qualitative studies only was not used. The reference lists of relevant studies were also hand searched to identify further relevant studies in line with best practice [16–18]. There is debate as to whether exhaustive searches are necessary in reviewing qualitative research with a purposive sampling of studies being proposed by some [10].

### Determining the relevance of studies

All returned titles were firstly screened for relevance by two authors independently (LJC & DMcC) with discrepancies resolved through discussion with a third senior author (SJK). All clearly irrelevant titles were excluded. All abstracts for clearly relevant and potentially relevant studies were then screened in a similar way. Finally, full text articles were obtained for all studies that appeared to meet the eligibility criteria based on the abstract and checked by two authors independently (LJC & EC), with discrepancies resolved through discussion with a third senior author once again.

The following inclusion and exclusion criteria were set a priori.

#### Participants

The review considered studies that include patients aged at least 18 years with capacity to consent for themselves and healthcare clinicians involved in the informed consent process.

#### Phenomena of interest

Included studies have explored patient’s or doctor’s experiences, attitudes or beliefs in relation to the informed consent process and doctor patient dialogue regarding treatment decisions. Studies examining ways in which doctors and patients believe the informed consent process could be improved or re-imagined were also included.

#### Context

Included studies involved patients and clinicians engaged in a consent; treatment decision; information giving process for a surgical or other invasive health care procedure. No limits were applied in terms of social, cultural, or economic context so the findings of this review might be widely applicable internationally.

#### Study types

Studies using qualitative methods of data collection and analysis were eligible for inclusion. This review considered studies including, but not limited to, designs such as phenomenology, grounded theory, ethnography, qualitative description, action research and feminist research. Many studies while appearing to employ qualitative methods are actually a quantitative tallying of how often certain themes are mentioned and as such there has been no qualitative interpretation of the data. Such studies have been excluded.

#### Exclusion criteria

Studies that explored issues related to proxy consent, for example, a parent consenting for a child or a family member consenting for an adult lacking capacity were excluded as we believe the decision-making process and information needs differ significantly between these situations and when patients are consenting for themselves. Similarly, studies evaluating the quality of consent for inclusion in clinical trials have been excluded, again because the motivation for trial participation is significantly different from that for patients consenting to surgery. Furthermore, this phenomenon is being investigated elsewhere [19].

### Appraisal of the quality of relevant studies

Relevant full text papers were analysed by two authors independently (LJC & EC). Each study was scored for quality according to the Joanna Briggs Institute (JBI) qualitative research appraisal tool [20]. (See Table 1) Studies were not excluded on quality grounds but low quality studies were further reviewed to assess the extent they altered the synthesis.

**Table 1:** JBI-QARI Critical Appraisal Tool

1. Is there congruity between the stated philosophical perspective and the research methodology?	
2. Is there congruity between the research methodology and the research question or objectives?	
3. Is there congruity between the research methodology and the methods used to collect data?	
4. Is there congruity between the research methodology and the representation and analysis of data?	
5. Is there congruity between the research methodology and the interpretation of results?	
6. Is there a statement locating the researcher culturally or theoretically?	
7. Is the influence of the researcher on the research, and vice-versa, addressed?	
8. Are participants, and their voices, adequately represented?	
9. Is the research ethical according to current criteria or, for recent studies, and is there evidence of ethical approval by an appropriate body?	
10. Do the conclusions drawn in the research report flow from the analysis, or interpretation, of the data?	

### Data extraction

Each relevant full text paper was analysed independently by two authors. Key details for each study were recorded including year of publication, country in which the study was conducted, the phenomenon of interest, context, qualitative method used, number of participants and cultural setting. Furthermore, note was made of funding sources and any potential conflicts of interest. Findings were defined as the main themes or findings detailed in the results section of each paper. Findings were extracted verbatim from the text of the study with an accompanying illustrative quote where available. Each finding was graded according to how confident the reader could be in that finding from the published data. Unequivocal findings are those that are matter of fact, beyond reasonable doubt, directly reported or observed and not open to challenge. Credible findings are plausible interpretations of the primary data within the selected theoretical framework. However, since they are interpretations of the primary data, they are open to challenge. Finally, unsupported findings are those with no evidence from the primary data [10, 20].

### Data synthesis

Findings were read repeatedly before being coded into categories of similar findings using NVivo qualitative data analysis Software; QSR International Pty Ltd. Version 12, 2012. Category descriptions were defined through consensus among the authors. These categories were reviewed and refined using a constant comparison method. Following classification of categories, synthesised findings combining two or more categories were agreed and developed through consensus among the authors.

## Results

**Fig. 1:**
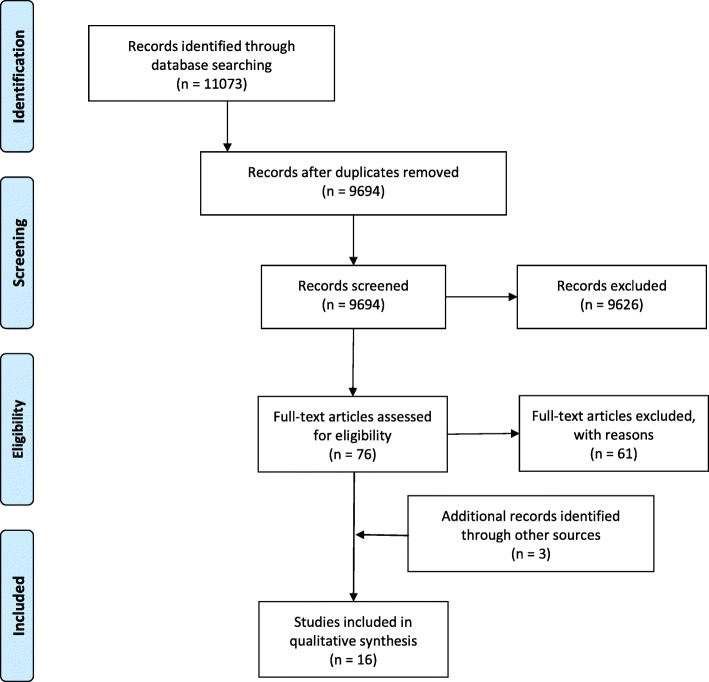
Prisma flow diagram - Identification of relevant studies

Searches of the bibliographic databases using the search strategy returned 11,073 titles. When duplicate results were removed, 9692 titles remained for screening. Of these, 74 full text articles went on to be assessed for eligibility. Sixty-one of these studies were excluded from the meta-synthesis because they did not meet inclusion criteria. Citations and reasons for exclusion for these studies are included in Additional File 2. The majority of papers excluded were not truly qualitative (*n* = 45). While many studies employed qualitative methods of data collection, they actually did not perform any qualitative analysis and simply reported quantitative measures of how often participants responded with a particular answer (n = 4). Thirteen studies met the eligibility criteria for inclusion in the meta-synthesis. A further 3 studies were identified through hand searching reference lists of included studies or because of prior knowledge of a relevant study, resulting in a total of 16 eligible articles. This process is summarised in the PRISMA [21] flow diagram in Fig. 1.

### Study characteristics

Included studies were published between 1996 and 2016. Most were conducted in the United Kingdom (UK) (*n* = 6) [22–27] or Canada (*n* = 5) [28–32] followed by the United States of America (USA) (*n* = 3) [33–35]. One study each was conducted in Norway and India. The 16 reports included the views of a wide variety of patients and healthcare professionals. Most studies examined patients in the post-operative period following elective surgery. However, some reports (n = 6) also included patients treated in an emergency setting [23–25, 27, 30, 36]. Six of the included studies explored patients’ or doctor’s views of the consent process for major or life-threatening procedures or conditions [26, 28, 30, 31, 33, 37]. Reports focused on patient perception of the informed consent process (*n* = 4) [24, 26, 31, 36], doctor’s perception of informed consent (*n* = 3, 25, 30, 36], patient’s preferences in making healthcare decisions (n = 4) [29, 34, 35, 37] and patient’s patterns of information seeking (*n* = 2) [28, 32]. One study each focused on the effectiveness of conveying consent information [33], the patient experience of surgery and surgeons [27], why some patients consent to surgery despite having reservations [23] and women’s views on a specific procedure (diagnostic laparoscopy) [22] as the main phenomenon of interest. These results are summarised in Table 2.

**Table 2:** Characteristics of included studies

Author	Year	Country	Setting	Emergency / Elective	Nature of procedure*	Population	Phenomena of Interest	Sample Size / Gender
**Berman**	2008	USA	Multi-Centre	Elective	Major	Patients with asymptomatic AAA.	Information important to patients facing healthcare decision. Evaluating how effective that information was conveyed.	20 M = 17 F = 3
**Bramall**	2014	Canada	Single Centre	Elective	Major	Post-operative neurosurgery patients with benign and malignant brain tumours	Patterns of information seeking. Suggestions for information provision.	31 M = 12 F = 19
**Dixon-Woods**	2006	UK	Single Centre	Elective and Emergency	Intermediate / Minor	Post-operative women following obstetrics and gynaecology surgery	Why some women sign consent forms even when they do not wish to consent to surgery or sign despite having reservations.	25 F = 25
**Habiba**	2004	UK	Single Centre	Elective and Emergency	Intermediate / Minor	Post-operative women following obstetrics and gynaecology surgery	The process of giving consent.	25 F = 25
**Hall**	2012	USA	Single Centre	Elective	Intermediate / Minor	Patients with inguinal hernia or benign biliary disease.	How patients make decisions through the process of informed consent	38 *Gender not recorded
**Kumar**	2012	India	Single Centre	Elective and Emergency	Intermediate / Minor	Patients and health care professionals in a surgical department.	Patient and doctor perceptions of informed consent, constraints to obtaining informed consent and their suggestions for improvement	14 Patients M = 6 F = 8 8 Doctors M = 6 F = 2
**McKneally**	2000	Canada	Single Centre	Elective	Major	Post-operative patients following oesophagectomy for oesophageal cancer	What patients believe about consent and decision making	36 M = 28 F = 8
**McKneally**	2004	Canada	Single Centre	Elective	Intermediate / Minor	Post-operative patients following laparoscopic cholecystectomy for gallstones	Patients perspective of the informed decision-making process	33 M = 13 F = 20
**McKneally**	2009	Canada	Single Centre	Elective and Emergency	Intermediate / Minor / Major	Attending (consultant) general and thoracic surgeons	Surgeons views of informed decision-making and consent	46 surgeons Thoracic = 28 General = 18
**McNair**	2016	UK	Multi-Centre	Elective	Major	Patients with oesophageal adenocarcinoma or squamous cell carcinoma facing surgery	Verbal information provision by surgeons during pre-operative consultations, and patient preferences for information about oesophageal cancer surgery.	31 M = 24 F = 7
**Meredith**	1996	UK	Multi-Centre	Elective and Emergency	Intermediate / Minor	Post-operative general surgery and urology patients	Patients experience of surgery and surgeons	30 Gender not recorded
**Moore**	2002	UK	Single Centre	Elective	Intermediate / Minor	Patients on a waiting list for a diagnostic laparoscopy	Women’s views of the risks and benefits of diagnostic laparoscopy in the investigation of chronic pelvic pain.	20 F = 20
**Schaufel**	2009	Norway	Single Centre	Elective	Major	Pre-operative patients for high risk PCI and cardiac surgery	Existential challenges of doctor-patient interaction and decision-making processes	10 M = 8 F = 2
**Spector**	2010	Canada	Multi-Centre	Elective	Intermediate / Minor	Post-operative plastic surgery patients	Expectations and informational needs of women who underwent three different breast procedures.	48 F = 48
**Suarez-Almazor**	2010	USA	Single Centre	Elective	Intermediate / Minor	Patients with a diagnosis of knee OA and no previous knee replacement	Decision making factors influencing patient preferences for TKA.	37 M = 14 F = 23
**Wood**	2014	UK	Multi-Centre	Elective / Emergency	Intermediate / Minor / Major	Consultant and training grade doctors from a range of surgical specialties.	Doctors’ perspectives of the informed consent process: how doctors communicate risk, barriers doctors face in gaining informed consent for surgical procedures, and how the current informed consent process can be improved.	20 M = 10 F = 10

### Participant characteristics

Included studies reported the views of 367 patients and 74 healthcare providers. From the 12 studies in which patient gender was reported, 38.1% (122/320) were male. Data on educational attainment and occupation was available for 47.1 and 27.5% of patients, respectively. The largest subgroup of included clinicians were male at consultant (attending) level. These results are summarised in Table 3.

**Table 3:** Participant characteristics

Total number of patients^a^	*n* = 398	
	Male *n* = 122(38.1%)	Female *n* = 198 (61.9%)
Highest level of educational attainment^b^	Less than High School	*n* = 22 (12.7%)
	High School	*n* = 48 (27.7%)
	College	*n* = 83 (48.0%)
	Professional Qualification	*n* = 20 (11.6%)
Occupation Status^c^	Employed	n = 45 (44.6%)
	Retired	*n* = 42 (41.6%)
	Home keeper	n = 10 (9.9%)
	Unemployed	n = 2 (2.0%)
	Student	n = 1 (1.0%)
	Disabled	n = 1 (1.0%)
Relationship Status	Single	n = 8 (7.9%)
	Married	*n* = 80 (79.2%)
	Separated/Divorced	n = 6 (5.9%)
	Widowed	*n* = 7 (6.9%)
Total number of clinicians^d^	*n* = 74	
	Male *n* = 16 (57.1%)	Female n = 12 (42.9%)
Level of training of clinicians	Consultant/Attending grade *n* = 57 (77.0%)	Training grade *n* = 17 (23.0%)

^a^Gender statistics available for 268 of 398 (67.3%) patients only

^b^ Educational attainment statistics available for 173 of 367 (47.1%) patients

^c^ Occupational and relationship status statistics available for 101 of 367 (27.5%) patients

^d^ Gender statistics available for 28 of 76 (36.8%) clinicians only

### Method of qualitative inquiry

The methods used in each of the included studies are summarised in Table 4. Most studies included in this review collected data through in-depth interviews(*n* = 13) [22–25, 27–34, 36]. Two reports use focus groups to collect participants insights [30, 35] while two studies generated data through observation of doctor-patient dialogue [26, 37].

**Table 4:** Qualitative methodology used in included studies

Author	Year	Method of Data Collection	Method of Analysis
Berman	2008	Face-to-face interviews	Constant comparison.
Bramall	2014	Face-to-face interviews	Constant comparison - NVIVO
Dixon-Woods	2006	Face-to-face interviews	Constant comparison – QSR N5
Habiba	2004	Face-to-face interviews	Constant comparison – QSR N5
Hall	2012	Telephone semi-structured interviews	Constant comparison
Kumar	2012	Face-to-face interviews	Framework analysis - NVIVO
McKneally	2000	Face-to-face interviews	Constant comparison – Ethnographic software
McKneally	2004	Face-to-face interviews	Constant comparison -QSR N5
McKneally	2009	Face-to-face semi-structured interviews and focus group discussions.	Constant comparison
McNair	2016	Observational study of doctor-patient dialogue and face-to-face interviews	Thematic analysis
Meredith	1996	Face-to-face interviews	Unclear
Moore	2002	Face-to-face interviews	Constant comparison
Schaufel	2009	Observational study of doctor-patient dialogue.	Discourse analysis and pragmatic linguistics
Spector	2010	Face-to-face interviews	Thematic analysis
Suarez-Almazor	2010	Focus group discussions	Constant comparison
Wood	2014	Face-to-face interviews	Thematic analysis - NVIVO

With regards the methods of qualitative analysis, the constant comparison technique was employed in most cases (*n* = 10) [22–24, 28–31, 33–35]. Thematic analysis was used in three instances [25, 26, 32] while framework analysis [36] and discourse analysis [37] were used in one study each, and the methods were unclear for the other study [27].

### Quality appraisal of included studies

All the included studies reported the use of accepted qualitative methods of data collection and analysis. Using the ten-point Joanna Brigg’s Institute qualitative appraisal checklist, all studies had evidence of congruity between the research methodology and the method of data collection, congruity between methodology and the interpretation of the results and the conclusions of each study flowed from the analysis of the data. Only five studies had included a statement to locate the researcher culturally or theoretically [22, 23, 26, 28, 36]. No studies were excluded based on quality. The outcome of the quality appraisal process is summarised in Additional File 3.

### Synthesised findings

Data extraction returned a total of 94 individual findings from the included studies. Following several rounds of coding and recoding, these findings were arranged into 17 categories. Those 17 categories were further organised into six synthesised findings. These synthesised findings were:

1. Inherent patient characteristics have a major impact on the conduct of the informed consent process.
2. Patients and doctors involved in the consent process described the transfer of knowledge as an important element of the consent process.
3. Consent was viewed as an exercise in communication skills by patients and clinicians.
4. Patients desire to be seen as a ‘model patient’ impairs their ability to actively participate in the informed consent process.
5. Trust could be built or diminished through the consent process. This trust influenced patient’s ultimate decision to consent or not to consent.
6. Decision making in patients engaged in the consent process was influenced by important other people, their physical condition and whether they perceived a choice of therapeutic options.

**Fig. 2:**
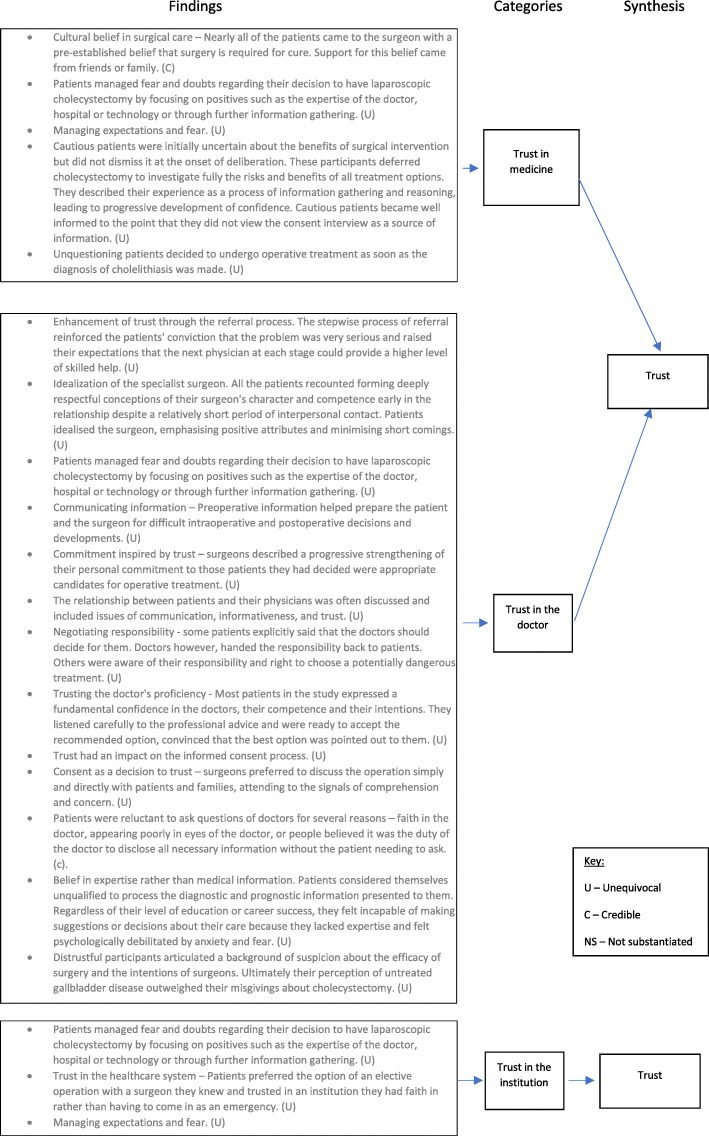
Trust synthesised finding

**Fig. 3:**
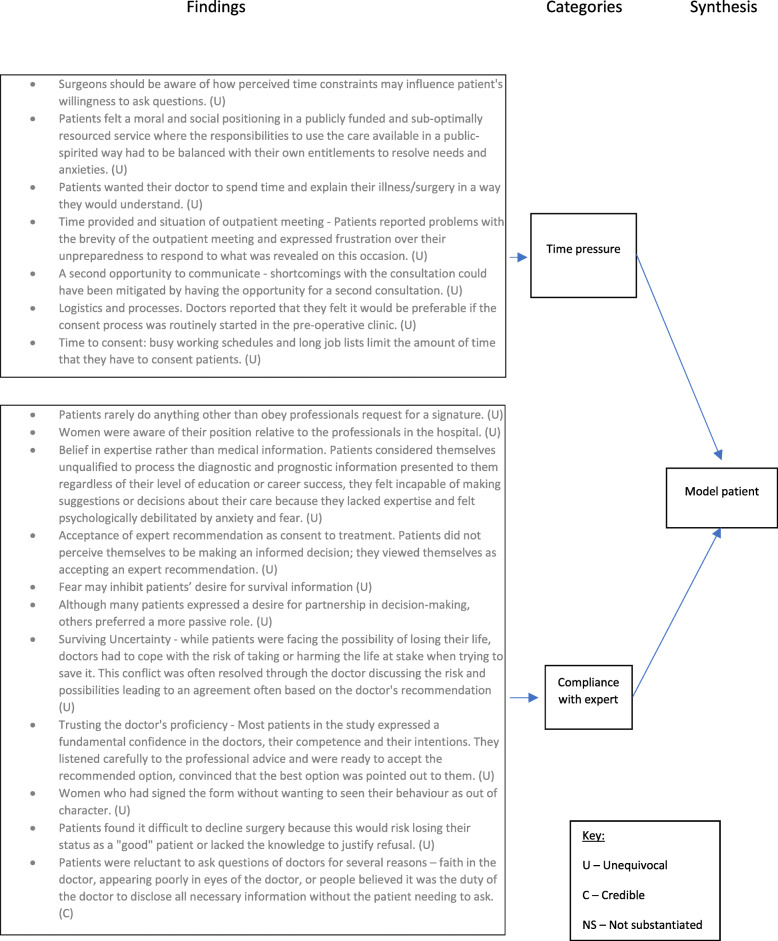
Model patient synthesised finding

Examples of the full synthesis for the trust and model patient synthesised findings are illustrated in Figs. 2 and 3, respectively. Similar figures for the remaining synthesised findings can be viewed in Additional Files 4, 5, 6 and 7.

#### Inherent patient characteristics have a major impact on the conduct of the informed consent process

Patients and clinicians included in the primary studies reported several inherent patient characteristics that had a significant influence on the informed consent process. This synthesised finding suggests that the ideal consent process is different for each patient dependent on their own set of characteristics.

##### Motivation for surgery

Several patients and clinicians discussed the fact that they often had a predetermined preference for a treatment option or a motivation for treatment before they engaged in the informed consent process. In total, nine findings from seven studies made up this category [22, 24, 29–31, 34, 35]. Several factors influenced a patient’s motivation for surgery including their previous experience of surgery or healthcare, believing that surgery was their only option (irrespective of whether that was truly the case) or a cultural belief in surgery.

**Table:** 

Finding	The majority of patients make or develop a clear preference for a decision before meeting a surgeon (U)
Illustration	“The doctor said I had a hernia, so I figured I have to go to the hospital to have it fixed.” [34]

##### Anxiety

In six studies, participants discussed how fear or anxiety impacted on their ability to participate in the informed consent process [25, 26, 30, 31, 35, 37]. Nine findings described how anxiety affected patient’s ability to both absorb information relayed during the consent process and ultimately make a decision. In some cases, patients and clinicians believed that initial anxieties could be in some way allayed or reduced through an effective consent process.

**Table:** 

Finding	Fear may inhibit patients’ desire for survival information (U)
Illustration	“I’ve got to ask the question because clearly those are the answers you want to know, you know. Am I gonna die? Or, you know, how long am I likely to live? You know, these are sort of basic questions that you want answers to but you’re scared that someone’s gonna say well, actually not very long’, you know (laughs) and you can’t argue because they’re the professional” [26]

##### Decision making style and information preferences

Fifteen findings extracted from 11 primary studies described issues relating to patients having different preferences for information and involvement in the decision-making process [22, 25–28, 30, 32–35, 37]. Some patients wished to be given all the details regarding their diagnosis, treatment options and the pros and cons of these options to enable them to have high levels of control in the decision-making process. However, others preferred not to be told about technical or risk related information and favoured decisions being made on their behalf by their doctor or others.

**Table:** 

Finding	All but one patient wanted to know about the risk of major complication so as to make her own judgement about the balance of risks. Knowing about this would have made them less frightened if a major complication did arise and allowed people to make appropriate contingency plans should a complication arise. (U)
Illustration	“If you woke up from the operation and you were expecting a little scar there and then all of a sudden they’re telling me I’ve got a massive scar and you’re thinking ‘well, why? What’s going on?’ You’re going to panic, aren’t you? - “Well I think you know, it’s always nice to weigh the benefits against the risk and then at least we could have made that as an informed decision that could have some serious consequences. I don’t think my husband and I, we hadn’t sort of prepared for all that, so it could have been fairly devastating.” [22]

##### Diagnosis/procedure

Six findings from four primary studies addressed how the health problem for which a patient was seeking treatment influenced the way in which the consent process was conducted or the patient’s ability to actively participate in that process [27, 28, 30, 31]. For example, patients and clinicians felt that patients consenting for a procedure related to a malignant diagnosis differed to patients receiving treatment for benign disease. The diagnosis seemed to influence patient’s perception of choice in the consent process and their anxiety levels.

**Table:** 

Finding	A malignant diagnosis changed information seeking behaviour (C)
Illustration	“patients were scared of the information they might find and found it anxiety provoking.” [28]

#### Patients and doctors involved in the consent process described the transfer of knowledge as an important element of the consent process

Almost every primary study included a finding related to the importance of the transfer of knowledge in the consent process. Three categories made up the knowledge synthesised finding. These were the concept of feeling inadequately informed in the consent process, that there exists an essential level of disclosure and that some patients sought out additional sources of information outside the consent consultation. Again, these findings speak to the consent process having a different meaning for individual patients. Some studies reported that poor levels of knowledge hampered patient’s ability to participate in the process while others claimed that they did not wish to have large volumes of information. In instances where knowledge was not a priority to patients, greater emphasis was placed on the quality of the doctor-patient relationship.

##### Feeling inadequately informed

Patients and clinicians reflected on several aspects of lacking the requisite knowledge to participate in the informed consent process in a meaningful way. There were several factors that influenced knowledge levels. These included the time allotted to the consent process, the patient’s desire for information and the expertise of the clinician in either having the knowledge or explaining it in a way that could be understood. Thirteen findings were extracted from ten primary studies. Patients described knowledge in terms of their understanding their condition, the treatment options available to them and what they ought to expect from their chosen option. No reference was made to recall of specific consent related details as being personally important to patients in the included studies.

**Table:** 

Finding	Patients were not adequately informed prior to making the decision (U)
Illustration	“I don’t know how they did it…the not knowing what’s going on is kind of difficult to handle” [33]

##### Essential level of disclosure

Seventeen findings extracted from eight sources were categorised as relating to patients or clinicians feeling that some information is essential to disclose within the informed consent process [22, 25, 26, 29–32, 36]. Doctors and patients described discomfort around discussing topics such as mortality within the consent process but acknowledged that such issues needed to be addressed. Many studies reported that initial uncertainty about choosing a surgical treatment was overcome through the transfer of this essential knowledge. Patients expected doctors to disclose all necessary information without them needing to ask additional questions or probe further.

**Table:** 

Finding	Survival information was desired by patients (U)
Illustration	“I’d like to know is- is your thoughts on, erm- on whether you’d like to know the- the chances of a successful cure and these kinds of things.” [26]

##### Additional sources of information

Patients and doctors discussed the sources of information that patients accessed in addition to the consent consultation. The internet, written information leaflets and discussions with friends and family were the most common resources employed. From the six primary studies included, additional information sources were sought out by patients who desired high levels of information as opposed to seeking out additional information where the consent process itself has been deficient [27–29, 32, 34, 35]. However, it appears that these additional information sources reinforced the message patients had heard from their clinicians and increased their confidence in the decision making process. Patients suggested that it was difficult for most lay people to discriminate good sources of information from less reliable ones and that doctors should routinely provide or guide patients to appropriate sources of additional information when it is desired.

**Table:** 

Finding	The role of printed information in communication - patients expressed an interest in more written materials. (C)
Illustration	“reading about it made me feel more positive and in control.” [27]

#### Consent was viewed as an exercise in communication skills by patients and clinicians

Fourteen findings from nine primary studies contributed to this synthesised finding. Patients and doctors discussed the importance of clear communication within the consent process. Patients and clinicians acknowledged that there was a certain amount of process and box ticking involved in the consent process and while many viewed this as bothersome, they believed it was unavoidable.

##### Clarity and free from jargon

Patients discussed that they desired information to be explained clearly to them in a way they understood and free from technical medical terms. Patients felt that failure to communicate important information in a way they understood impaired their ability to participate in the decision-making process and heightened fear and anxiety. Clinicians reflected that they struggled to communicate the uncertainty around the outcomes from surgery to patients and in practice they often presented technical information with little explanation. Nine findings from seven primary studies were included in this category [22, 25–28, 31, 36].

**Table:** 

Finding	Surgeons should be forthright with information and avoid medical jargon (U)
Illustration	“I never remember them calling it a tumour… the language doctors use is different than people use normally, day to day.” [28]

##### Process

People discussed the process of consent in five different findings extracted from four studies [24–26, 30]. Consent served a number of different purposes: as a legal entity, an educational process and as a ritual achieved through doctor-patient communication. Both patients and clinicians commented that they felt that consent was in the most part a ritualistic information communicating process but was necessary from a legal perspective and that information had to be communicated to patients (even when they said they did not want to hear it).

**Table:** 

Finding	There were a range of meanings given to the consent form (U)
Illustration	As a legal entity “I think to cover themselves. If anything did go wrong you know, you were, you were signing to say… you’ve accepted the …risks that were involved… The consent form as a ritual - “All I remember is that it being shoved under my nose and saying you’ve got to go down to surgery, sign and that was it” The consent forma as control and power -"It’s only when the consent form comes that you have got the choice to turn around and say you don’t want it...” [24]

#### Patient’s desire to be seen as a ‘model patient’ impairs their ability to actively participate in the informed consent process

Patients discussed a sense of fear or apprehension that their behaviour or actions might be seen as deviating from what would be considered that of the ‘model patient’. Model patient actions included not disagreeing with the clinician, not questioning expert advice and not ‘wasting’ the doctor’s time with trivial queries. These findings are summarised in Fig. 2.

##### Time pressure

Patients felt a responsibility not to waste doctor’s time with, what they thought were, their own trivial queries in relation to consent. These feelings were heightened where patients were using publicly funded and resource strapped healthcare. In such cases, patients felt a need to use healthcare in a public spirited fashion being aware of the needs of the patients in the waiting room [23]. Seven findings from five studies reported the importance of the time taken to conduct the consent process [23, 25, 27, 28, 36]. Doctors discussed time pressure was a significant barrier in their ability to impart as much information as they would like resulting in the patient agreeing to treatment despite not being optimally informed.

**Table:** 

Finding	Patients felt a moral and social positioning in a publicly funded and sub-optimally resourced service where the responsibilities to use the care available in a public-spirited way had to be balanced with their own entitlements to resolve needs and anxieties. (U)
Illustration	“you think oh you [doctor] don’t got time to listen to me, you know what I mean, because you have got other patients waiting outside, you gotta think of them, you know what I mean, so I’m that sort of person, so no I would have liked to have asked now but it is too late now” [23]

##### Compliance with expert advice

A total of 11 findings from six primary studies related to the phenomenon of patients feeling that they should simply comply with the doctor’s recommendation for treatment [23, 26, 31, 35–37]. Patients discussed feelings of not wanting their behaviour to be seen out of character by questioning the doctor, that the consent form was simply signed as a matter of process with little thought and that some patients had agreed to treatment despite not understanding what they had agreed to. Patients explained such behaviour by having high levels of trust in the treating clinician expecting them to ‘know best’ or they felt that they lacked the knowledge to enable them to question their care.

**Table:** 

Finding	Patients found it difficult to decline surgery because this would risk losing their status as a “good” patient or lacked the knowledge to justify refusal. (U)
Illustration	“the last thing they need is someone turning around and saying I’ve changed my mind I don’t want to have this because it messes you know all their sort of thing up.” [23]

#### Trust can be built or diminished through the consent process. Trust influenced patients involved in the consent process ultimate decision

Trust was a predominant theme present in almost half of included studies. Patients and doctors discussed the importance of trust in; the doctor-patient relationship, in medicine as an entity and in the institution. Several factors enhanced or diminished the levels of trust that patients felt. This finding is summarised in Fig. 3.

##### Trust in medicine

**Table:** 

Finding	Unquestioning patients decided to undergo operative treatment as soon as the diagnosis of cholelithiasis was made. (U)
Illustration	“Once I knew what the problem was I really didn’t want to hear any argument about it. I just wanted the gallbladder out.” “He said, gallstones—they have to come out. I said fine, let’s take them out. It’s that simple; let’s do it.” [29]

Patients reported that they had a sense of trust in the medical profession, medical science and the technology of surgery that influenced their decision making in the consent process. Five findings from three primary studies, all written by McKneally and colleagues, were included in this category [29–31]. Both doctors and patients recounted experiences whereby patients exhibited unwavering faith in surgery as a solution to their medical condition. This resulted in these patients paying little attention to risk information and alternatives of surgical care. Some patients believed that the treatment was right for them and the consent process did nothing to change their mind.

##### Trust in the institution

Two primary studies, again both from McKneally and colleagues, contributed to this category [29, 30]. Patients managed their anxieties relating to surgery by focusing on the positive reputation of the institution that they were being treated in. Doctors used the results of the institution they were working in to allay patient fears and increase patient trust in the healthcare facility. Patients expressed a preference for planned treatment in an institution that they had faith in rather than having to come in as an emergency and lose control over where and who would be caring for them.

**Table:** 

Finding	Patients managed fear and doubts regarding their decision to have laparoscopic cholecystectomy by focusing on positives such as the expertise of the doctor, hospital or technology or through further information gathering. (U)
Illustration	“He is a very, very good doctor. Very smart. I trust him....” “...I thought at least this is the place that all my regular doctors recommend” “I had to give in... by the time so many people told me that the laparoscopic procedure was simple, I swallowed my reluctance.” [29]

##### Trust in the doctor

Trust in the individual clinician undertaking the consent process was cited as an important factor within the consent process by patients and clinicians in seven primary studies [29–31, 33, 35–37]. Patients reported that this trust was built through good communication, a sense that the doctor had their best interests at heart, through the recommendation of friends, family or their family doctor and in the doctor’s interpersonal skills. Patients reported feeling that their doctor would tell them all the information they required to make an informed choice. Meanwhile doctors reported that building trust in the consent process was important if there were complications as a result of the surgery. Trust built in the preoperative period reduced the chances of the patient losing faith in their doctor when things did not go as planned.

One primary study also included a finding that some patients were initially distrustful of surgeons but ultimately went ahead with surgical treatment as leaving their condition untreated outweighed their misgivings about surgery. It appears, from some of the included studies, that patient trust in the individual doctor had a greater bearing on their decision to consent to treatment than the communication of consent related knowledge had.

**Table:** 

Finding	Trusting the doctor’s proficiency - Most patients in the study expressed a fundamental confidence in the doctors, their competence and their intentions. They listened carefully to the professional advice and were ready to accept the recommended option, convinced that the best option was pointed out to them. (U)
Illustration	Helen (P): “Yes, no, you decide, I won’t say anything.” Luke (D): “No, it is a difficult—” Helen (P): “It’s got to be the ones who understand it.” (…) Luke (D): “If I understand you properly, then you are willing to undergo surgery if we think it’s medically—” Helen (P): “Yes. Exactly. You are the ones to decide.” Luke (D): “Yes—we can only give advice and recommendation.” Helen (P): “Yes, yes, of course.” Luke (D): “And in the end it’s for you to decide.” [37]

#### Decision making in patients engaged in the consent process was influenced by important other people, their physical condition and whether they perceived a choice of therapeutic options

Several themes emerged that related to decision making within the consent process. Firstly, patients discussed the role that other people (besides the doctor) had in helping them arrive at their decision to consent. Secondly, patients felt that, in many cases they did not feel that they had a realistic choice in the consent process, sensing that surgery was their only option. Finally, the primary studies reported findings relating to the impact that a patient’s current physical state had on their ability to engage in the consent and decision-making processes.

##### Important others

Three findings from three individual studies were categorised as relating to the role of important other people who assisted patients in the decision making process [23, 32, 34]. The authors of these studies discussed that patients in hospital are often removed from their usual social constructs, relying on the input of friends and family who accompany them. It appears that most patients included in these studies valued the support of friends and family in assisting them with their decision making in relation to consent.

**Table:** 

Finding	82% identified at least one or more people who helped them make their decision for surgery. (U)
Illustration	“well, I had my mother here and she was like, well, I do not want one [gallstone] to get stuck and you to get jaundice and stuff” [34]

##### Choice

Many patients felt that the options available to them for treatment of their medical problem was purely illusory. For some, in the emergency setting or those with a malignant diagnosis they felt that surgery was their only realistic treatment option. The presentation of alternatives was irrelevant as they would rather accept the risk of surgery instead of facing a miserable death if they did not undergo surgery. Some patients’ faith in surgical treatment meant that despite there being a range of surgical and non-surgical options available to them that they did not consider these as serious choices. Patients felt that in some instances they lacked the requisite knowledge to be able to evaluate their options in a meaningful way and were happy to be guided by the clinician as to what to choose. This category was made up of 12 findings extracted from seven studies [22, 23, 31, 33–35, 37].

**Table:** 

Finding	While most patients perceive a choice to have surgery or not many see it as a necessity. (U)
Illustration	“There is no choice: you either have the surgery or you have this for the rest of your life… I knew right away that I had to, you know, I had to have the surgery” [34]

##### Physical state

Three findings from two studies reported that factors such as pain, fear, anxiety and medications all had a significant impact on the patients ability to fully engage in the informed consent and decision-making processes [23, 31]. In these cases, patients deferred the decision making to the doctor and while they may have indicated their consent, it was by no means informed.

**Table:** 

Finding	Women felt their capital [to make a decision] was seriously diminished by a number of features of the situation e.g. pain, drugs, extreme states. (U)
Illustration	“I think the pain was taking over, I don’t think I was completely in, and I was on morphine anyway, I was having gas and air so I don’t think I was completely compos mentis as such” [24]

## Discussion

Informed consent for clinical procedures is a complex process involving the exchange of information from clinicians to patients, in which patients process information before making a decision that is free from coercion. Existing research that has investigated methods of improving informed consent has focused on improving patient knowledge without unduly increasing patient anxiety levels at the time of consent. However, this review demonstrates that while all aspects of knowledge are clearly an important element to patients and their doctors it does not reflect the complexity of the consent process.

Almost all included studies included a reference to patient knowledge as an important element of the consent process. Knowledge in the studies included in this review has referred to understanding consent related information such as the options available to the patient and the potential harms and benefits associated with each of those options. This is in stark contrast to the existing body of consent research which has focused on objective measures of patient recall in determining the success or failure of the consent process [6]. In fact, within the included studies, patients stated that “feeling” informed and understanding was most important to them rather than being given specific details [26, 36]. These findings suggest that future research evaluating interventions to improve informed consent for surgery ought to focus on trying to evaluate patient understanding as opposed to simply focusing on the much more easily captured metric of recall or the volume of information provided.

This review suggested also that it was difficult for most patients to discriminate good sources of information from less reliable ones. Further the language used in many patient educational tools may be difficult for many to comprehend [38].

A measure of trust has not been incorporated into any consent related research previously. In half of the studies in this review, patients and doctors discussed the importance of trust in the doctor-patient relationship, the patient and the institution and the patient and medicine in general. It may be the case that researchers believe that trust is too difficult a concept to measure in a trial setting. Despite this, there are at least three validated tools designed to capture the level of inter-personal trust between a patient and their doctor at a given moment in time [39–41]. It may also be the case that people believe that consent in current medical practice is obsolete and even dangerous. Such thoughts have been reinforced by high profile inquiries such as those at the Bristol Royal Infirmary [42] and Alder Hey [43] where trust in the medical profession seems to have been abused.

Manson and O’Neill [44] argue in their seminal text, that the informed consent process encompasses more than just the transfer of knowledge (the “speech content”), it also encompasses what is done (the “speech act”). They contest that truly informed consent is rarely possible because patients may not have the capacity to fully comprehend the complexity of the decision they are making and even when they do possess the capacity their capital to fully participate in the consent process is diminished by their physical state or anxiety. While they explicitly state that they do not advocate a return to paternalism, their argument again emphasises the importance of trust within the consent process and points to a measurement of trust being reflective of the quality of the informed consent process.

This review highlights themes relating to decision-making in the consent process. Patients did not perceive a sense of choice in the consent process, which clearly flies in the face of bioethical principals relating to autonomy. While the shared-decision making literature has gone some way in addressing this issue, clearly more needs to be done before this knowledge is widely incorporated into everyday consent practices [45]. Patients highlighted the important contribution that trusted friends and family make in helping them arrive at a decision for treatment, yet this has not been investigated in existing consent literature.

Several primary studies contributed to the model patient synthesised finding. It was clear that patients occasionally complied with a treatment plan even when it did not align with their own beliefs and values or without feeling fully informed. Patients cited perceived pressures created by inadequate time for consent, their lack of knowledge and feeling that they did not want to be seen poorly in the doctor’s eyes as the key reasons for this behaviour. One of the key components of informed consent is that the patient’s choice is made free from coercion [46]. Although there is no evidence of direct coercion from the primary studies included in this review, factors such as inadequate time, social hierarchies and patients feeling inadequately informed may be indirectly leading to forced choices.

Several inherent patient characteristics were identified as being important covariates in determining the success or failure of the consent process. This included things such as anxiety, the diagnosis the patient is seeking medical treatment for and the patient’s desire for information and involvement in the process. Anxiety has been commonly investigated as an outcome when trialling interventions to improve informed consent [6]. While researchers have worried that enhanced information may heighten patient anxiety, this review suggests that baseline patient anxiety may inhibit their ability to participate in the consent process in the first instance. The review also clearly demonstrates that not all patients desire the same type and quantity of consent related information and clearly a one size fits all approach is not suitable. It may be the case that efforts ought to be made at the outset of the process to determine patients wants and desires rather than the level of information disclosure being determined by the medical profession, a point articulated by Manson and O’Neill too [44]. A solution to this problem may lie in the development of “Core Information Sets” [47]. These information sets, developed through consensus between patients, health care workers, patient family members and patient support groups, aim to present the essential information for a given diagnosis or procedure. Of course, some patients may not wish to hear any consent related information but at least core information sets go some way to determining the information which is deemed important by both clinicians and their patients.

This review is not without some limitations. As previously discussed, the indexing of qualitative literature is notoriously inconsistent with many of the major databases only beginning to properly catalogue such research as qualitative in recent years [16]. We attempted to overcome this problem by casting our original search sufficiently wide to improve our chances of capturing all relevant titles. Despite this, three of the included studies came from checking the reference lists of included studies. As such, other relevant studies may exist but have not been captured by our search.

We have only included studies written in English for this review. While no non-English studies were identified through our searches, it may be the case that if the search strategy had been written in other languages it would have yielded relevant studies that were not forthcoming because of the indexing problems previously discussed. Furthermore, only one study [36] was from a non-westernised society. This study makes it clear that the cultural and societal norms surrounding consent differed from the western view of consent. There is considerable evidence that consent practices differ throughout the world with open discussion about adverse events and death being deemed as harmful in some societies [48]. As such, this review only truly reflects the opinions of patients and clinicians in the societies like the UK, USA and Canada.

## Conclusion

This qualitative evidence synthesis has, for the first time, systematically catalogued and organised the findings of existing qualitative research pertaining to the informed consent process. It highlights the elements of consent that are important to both doctors and patients and demonstrates the gaps between the outcomes that have traditionally been measured in consent research and perhaps should be measured going forward. This qualitative evidence synthesis will inform the next stage in the development of a COS that should be used to evaluate the effects of interventions designed to improve informed consent for surgery.

## Supplementary information

**Additional file 1.**

**Additional file 2.**

**Additional file 3.**

**Additional file 4.**

**Additional file 5.**

**Additional file 6.**

**Additional file 7.**

## Data Availability

All data generated or analysed during this study are included in this published article [and its supplementary information files].

## Abbreviations

PROSPERO: Prospective Register for Systematic Reviews
PRISMA: Preferred Reporting Items for Systematic Reviews and Meta-Analyse.
LJC: Liam Convie
EC: Elaine Carson
DMcC: Darren McCusker
RSMcC: Scott McCain
NMcK: Nicola McKinley
WJC: Jeffrey Campbell
MC: Mike Clarke
SJK: Stephen J Kirk
