# Supplementary material for: The patient and clinician experience of informed consent for surgery: a systematic review of the qualitative evidence
Source: BMC Med Ethics. 2020 Jul 11;21:58. doi: 10.1186/s12910-020-00501-6 (PMC7353438; doi:10.1186/s12910-020-00501-6)
Supplement: Supplementary file 1 — Additional file 1. [file 12910_2020_501_MOESM1_ESM.docx]

Database(s): **Embase**1974 to 2017 Week 37 
Search Strategy:

| **#** | **Searches** | **Results** |
| --- | --- | --- |
| 1 | informed consent/ | 87102 |
| 2 | ((written or verbal or process*) adj5 consent*).mp. [mp=title, abstract, heading word, drug trade name, original title, device manufacturer, drug manufacturer, device trade name, keyword, floating subheading word] | 20366 |
| 3 | patient decision making/ | 8394 |
| 4 | "decision aid*".mp. [mp=title, abstract, heading word, drug trade name, original title, device manufacturer, drug manufacturer, device trade name, keyword, floating subheading word] | 3085 |
| 5 | exp patient attitude/ | 333649 |
| 6 | "patient* choice*".mp. [mp=title, abstract, heading word, drug trade name, original title, device manufacturer, drug manufacturer, device trade name, keyword, floating subheading word] | 3896 |
| 7 | patient autonomy/ | 4560 |
| 8 | 1 or 2 or 3 or 4 or 5 or 6 or 7 | 433800 |
| 9 | exp surgery/ | 4177284 |
| 10 | (surger* or "surgical procedure*").mp. [mp=title, abstract, heading word, drug trade name, original title, device manufacturer, drug manufacturer, device trade name, keyword, floating subheading word] | 3167096 |
| 11 | (invasive adj3 procedure*).mp. [mp=title, abstract, heading word, drug trade name, original title, device manufacturer, drug manufacturer, device trade name, keyword, floating subheading word] | 68596 |
| 12 | exp endoscopy/ | 530826 |
| 13 | lumbar puncture/ | 18027 |
| 14 | 9 or 10 or 11 or 12 or 13 | 5100194 |
| 15 | information dissemination/ | 18103 |
| 16 | consumer health information/ | 3249 |
| 17 | health literacy/ | 6630 |
| 18 | patient education/ | 101058 |
| 19 | teach-back.mp. [mp=title, abstract, heading word, drug trade name, original title, device manufacturer, drug manufacturer, device trade name, keyword, floating subheading word] | 252 |
| 20 | exp interpersonal communication/ | 521616 |
| 21 | human relation/ or doctor patient relation/ or nurse patient relationship/ | 228533 |
| 22 | audiovisual aid/ | 579 |
| 23 | book/ | 35801 |
| 24 | medical illustration/ | 3425 |
| 25 | multimedia/ | 3215 |
| 26 | videorecording/ or videotape/ | 66379 |
| 27 | (computer* adj3 (instruction* or teach* or educat*)).mp. [mp=title, abstract, heading word, drug trade name, original title, device manufacturer, drug manufacturer, device trade name, keyword, floating subheading word] | 3364 |
| 28 | social support/ | 75023 |
| 29 | intervention*.mp. [mp=title, abstract, heading word, drug trade name, original title, device manufacturer, drug manufacturer, device trade name, keyword, floating subheading word] | 1105876 |
| 30 | 15 or 16 or 17 or 18 or 19 or 20 or 21 or 22 or 23 or 24 or 25 or 26 or 27 or 28 or 29 | 1966572 |
| 31 | 8 and 14 and 30 | 22582 |
| 32 | limit 31 to (human and english language) | 20331 |
| 33 | limit 32 to (adult <18 to 64 years> or aged <65+ years>) | 9151 |
| 34 | exp mental health/ | 119690 |
| 35 | exp mental disease/ | 1909094 |
| 36 | exp schizophrenia/ | 169375 |
| 37 | 34 or 35 or 36 | 1967115 |
| 38 | 33 not 37 | 8085 |
| 39 | (improv* or enhanc* or increas* or standardi#e*).mp. [mp=title, abstract, heading word, drug trade name, original title, device manufacturer, drug manufacturer, device trade name, keyword, floating subheading word] | 8781100 |
| 40 | 38 and 39 | 4254 |
| 41 | limit 40 to embase | 3238 |

Database: PsycINFO <1806 to September Week 1 2017>

Search Strategy:

--------------------------------------------------------------------------------

1 exp Informed Consent/ (3917)

2 (written consent or verbal consent or consent process or decision aid).mp. [mp=title, abstract, heading word,

table of contents, key concepts, original title, tests & measures] (1472)

3 exp Client Attitudes/ or patient acceptance of healthcare.mp. (19892)

4 exp Client Satisfaction/ (4944)

5 exp preferences/ (26419)

6 exp Treatment Refusal/ (704)

7 exp decision making/ (92390)

8 exp Choice Behavior/ or client* choice.mp. (25167)

9 or/1-8 (138420)

10 exp DENTAL SURGERY/ or exp PLASTIC SURGERY/ or exp BARIATRIC SURGERY/ or exp HEART SURGERY/ or exp ENDOCRINE

GLAND SURGERY/ or exp SURGERY/ (52030)

11 (invasive adj3 (procedure* or intervention* or process* or examination*)).mp. [mp=title, abstract, heading word,

table of contents, key concepts, original title, tests & measures] (999)

12 endoscopy.mp. (311)

13 lumbar puncture.mp. (914)

14 or/10-13 (53996)

15 exp Information Dissemination/ (1461)

16 health education/ or exp client education/ or exp health knowledge/ or exp health literacy/ (22604)

17 exp Client Education/ (3568)

18 exp Teaching Methods/ or exp Educational Audiovisual Aids/ or exp Learning/ or exp Computer Assisted Instruction/

(249478)

19 educational audiovisual aids/ or exp audiovisual communications media/ or exp instructional media/ or exp

videotape instruction/ (31951)

20 books/ or reading materials/ (4559)

21 exp INTERVENTION/ (87650)

22 exp "TOOL USE"/ (1251)

23 or/15-22 (385052)

24 9 and 14 and 23 (140)

***************************

Cochrane Library Search 14^th^ September 2017

#1 MeSH descriptor: [Informed Consent] explode all trees 638

#2 (informed near/3 (consent or decision or choice)):ti,ab,kw (Word variations have been searched) 9684

#3 (consent near/3 (process or form or document or written or verbal)):ti,ab,kw (Word variations have been searched) 3295

#4 #1 or #2 or #3 10302

#5 MeSH descriptor: [Specialties, Surgical] explode all trees 1983

#6 MeSH descriptor: [Endoscopes] explode all trees 1144

#7 MeSH descriptor: [Radiology, Interventional] explode all trees 44

#8 MeSH descriptor: [Spinal Puncture] explode all trees 283

#9 MeSH descriptor: [Disclosure] explode all trees 383

#10 MeSH descriptor: [Information Dissemination] explode all trees 235

#11 MeSH descriptor: [Patient Education as Topic] explode all trees 8200

#12 MeSH descriptor: [Communication] explode all trees 12633

#13 MeSH descriptor: [Audiovisual Aids] explode all trees 3359

#14 MeSH descriptor: [Computer-Assisted Instruction] explode all trees 1137

#15 MeSH descriptor: [Decision Support Techniques] explode all trees 3649

#16 (visual aid or decision aid or diagram or video or graph):ti,ab,kw (Word variations have been searched) 10996

#17 (audio-visual or videotape):ti,ab,kw (Word variations have been searched) 2517

#18 (information near/3 (leaflet or book or pamphlet or sheet or booklet or written)):ti,ab,kw (Word variations have been searched) 1340

#19 (enhance or improve or increase or standardise):ti,ab,kw (Word variations have been searched) 409022

#20 MeSH descriptor: [Decision Making] explode all trees 3728

#21 #9 or #10 or #11 or #12 or #13 or #14 or #15 or #16 or #17 or #18 #19 or #20 39690

#22 #4 and #21 1144

#23 surgery or invasive procedure (Word variations have been searched) 170186

#24 #5 or #6 or #7 or #8 or #23 171630

#25 #22 and #24 256

Medline Search 1946 – 13^th^ September 2017

| 1 | Informed Consent/ | 35076 |
| --- | --- | --- |
| 2 | "written consent*".mp. [mp=title, abstract, original title, name of substance word, subject heading word, keyword heading word, protocol supplementary concept word, rare disease supplementary concept word, unique identifier, synonyms] | 1452 |
| 3 | "verbal consent*".mp. [mp=title, abstract, original title, name of substance word, subject heading word, keyword heading word, protocol supplementary concept word, rare disease supplementary concept word, unique identifier, synonyms] | 216 |
| 4 | "consent process*".mp. [mp=title, abstract, original title, name of substance word, subject heading word, keyword heading word, protocol supplementary concept word, rare disease supplementary concept word, unique identifier, synonyms] | 1776 |
| 5 | "decision aid*".mp. [mp=title, abstract, original title, name of substance word, subject heading word, keyword heading word, protocol supplementary concept word, rare disease supplementary concept word, unique identifier, synonyms] | 1993 |
| 6 | "patient acceptance of health care"/ or patient satisfaction/ or patient preference/ or treatment refusal/ | 126552 |
| 7 | "patient* choice*".mp. [mp=title, abstract, original title, name of substance word, subject heading word, keyword heading word, protocol supplementary concept word, rare disease supplementary concept word, unique identifier, synonyms] | 2143 |
| 8 | "patient* autonomy*".mp. [mp=title, abstract, original title, name of substance word, subject heading word, keyword heading word, protocol supplementary concept word, rare disease supplementary concept word, unique identifier, synonyms] | 2337 |
| 9 | 1 or 2 or 3 or 4 or 5 or 6 or 7 or 8 | 165515 |
| 10 | exp Specialties, Surgical/ | 187958 |
| 11 | (surger* or "surgical procedure*").mp. [mp=title, abstract, original title, name of substance word, subject heading word, keyword heading word, protocol supplementary concept word, rare disease supplementary concept word, unique identifier, synonyms] | 1187683 |
| 12 | (invasive adj3 procedure*).mp. [mp=title, abstract, original title, name of substance word, subject heading word, keyword heading word, protocol supplementary concept word, rare disease supplementary concept word, unique identifier, synonyms] | 38889 |
| 13 | exp Endoscopy/ | 319314 |
| 14 | Spinal Puncture/ | 5919 |
| 15 | "lumbar puncture*".mp. [mp=title, abstract, original title, name of substance word, subject heading word, keyword heading word, protocol supplementary concept word, rare disease supplementary concept word, unique identifier, synonyms] | 6725 |
| 16 | 10 or 11 or 12 or 13 or 14 or 15 | 1517099 |
| 17 | Information Dissemination/ | 14153 |
| 18 | consumer health information/ or health literacy/ or patient education as topic/ or teach-back communication/ | 86178 |
| 19 | "interpersonal communication*".mp. [mp=title, abstract, original title, name of substance word, subject heading word, keyword heading word, protocol supplementary concept word, rare disease supplementary concept word, unique identifier, synonyms] | 1074 |
| 20 | professional-patient relations/ or nurse-patient relations/ or physician-patient relations/ | 126534 |
| 21 | audiovisual aids/ or books, illustrated/ or medical illustration/ or multimedia/ or videodisc recording/ or videotape recording/ | 25104 |
| 22 | Computer-Assisted Instruction/ | 11280 |
| 23 | community networks/ or social support/ | 69544 |
| 24 | intervention*.mp. [mp=title, abstract, original title, name of substance word, subject heading word, keyword heading word, protocol supplementary concept word, rare disease supplementary concept word, unique identifier, synonyms] | 716279 |
| 25 | 17 or 18 or 19 or 20 or 21 or 22 or 23 or 24 | 989331 |
| 26 | 9 and 16 and 25 | 4813 |
| 27 | limit 26 to (english language and humans) | 4257 |
| 28 | limit 27 to "all adult (19 plus years)" | 2859 |
| 29 | Mental Health/ | 30001 |
| 30 | exp mental disorders/ or exp schizophrenia/ | 1129832 |
| 31 | 29 or 30 | 1150060 |
| 32 | 28 not 31 | 2758 |

Web of Science Search Strategy 12^th^ September 2017


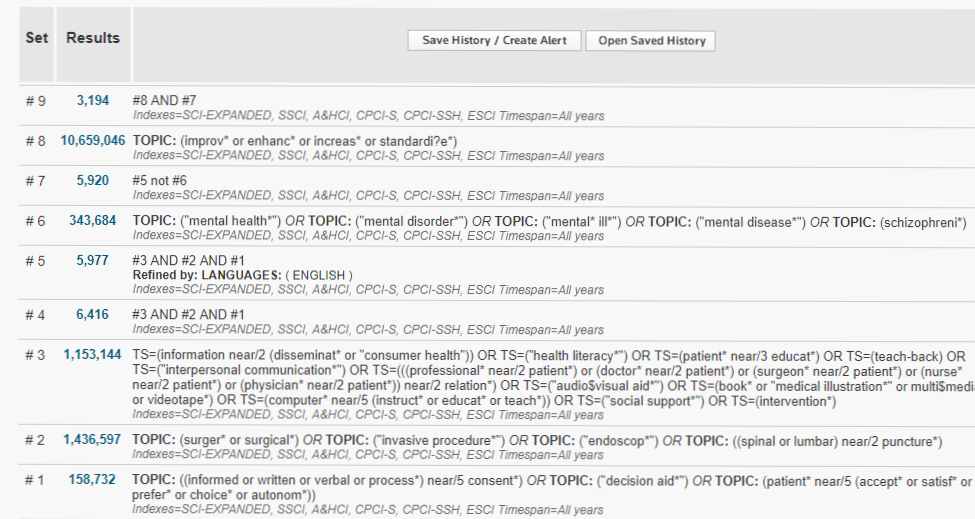


CINAHL Search 14^th^ September 2017

| **#** | **Query** | **Limiters/Expanders** | **Results** |
| --- | --- | --- | --- |
| S16 | S4 AND S9 AND S14 | Narrow by Language0: - english  Search modes - Boolean/Phrase | 1,487 |
| S15 | S4 AND S9 AND S14 | Search modes - Boolean/Phrase | 1,517 |
| S14 | S10 OR S11 OR S12 OR S13 | Search modes - Boolean/Phrase | 506,631 |
| S13 | TX visual aid* or decision aid* or diagram* or graph* or video* or video tape* | Search modes - Boolean/Phrase | 58,303 |
| S12 | TX (information N3 (leaflet* or pamphlet* or sheet* or written or verbal or audiovisual or av or videotape)) | Search modes - Boolean/Phrase | 3,186 |
| S11 | TX ( ((interactive ir communicat*) N3 tool* or intervention* or procedure*) ) OR TX interactive N3 comminicat* | Search modes - Boolean/Phrase | 456,214 |
| S10 | ( TX ( (consent N3 (enhanc* or improv* or increas*)) ) ) OR ( TX ( *consent N3 (method* or procedure* or obtain* or elicit*)) ) ) | Search modes - Boolean/Phrase | 4,574 |
| S9 | S5 OR S6 OR S7 OR S8 | Search modes - Boolean/Phrase | 497,144 |
| S8 | "interventional cardiology" | Search modes - Boolean/Phrase | 367 |
| S7 | (MH "Radiography, Interventional") | Search modes - Boolean/Phrase | 3,111 |
| S6 | (MH "Endoscopy+") OR (MH "Endoscopy, Gastrointestinal+") OR (MH "Endoscopy, Digestive System+") | Search modes - Boolean/Phrase | 63,796 |
| S5 | (MH "Surgery, Operative+") | Search modes - Boolean/Phrase | 462,869 |
| S4 | S1 OR S2 OR S3 | Search modes - Boolean/Phrase | 29,573 |
| S3 | TX ( (consent* N2 (process* or form* or document* or written or verbal)) ) | Search modes - Boolean/Phrase | 4,614 |
| S2 | TX ((informed N3 (consent or decision* or choice*)) | Search modes - Boolean/Phrase | 16,502 |
| S1 | (MH "Consent+") | Search modes - Boolean/Phrase | 15,201 |
