# Supplementary material for: The patient and clinician experience of informed consent for surgery: a systematic review of the qualitative evidence
Source: BMC Med Ethics. 2020 Jul 11;21:58. doi: 10.1186/s12910-020-00501-6 (PMC7353438; doi:10.1186/s12910-020-00501-6)
Supplement: Supplementary file 2 — Additional file 2. [file 12910_2020_501_MOESM2_ESM.docx]

| Author | Year | Reason for Exclusion |
| --- | --- | --- |
| Ashraf[1] | 2014 | Quantitative study |
| Bosworth[2] | 2004 | Quantitative study |
| Bowden[3] | 2004 | No qualitative analysis |
| Braddock[4] | 2008 | Quantitative study |
| Brosnam[5] | 2009 | Audit |
| Byrne[6] | 1988 | Quantitative study |
| Cawich[7] | 2013 | No qualitative analysis |
| Clarke[8] | 2007 | Opinion paper |
| Corda[9] | 2011 | Quantitative study |
| Courtney[10] | 2001 | Quantitative study |
| Dathatri[11] | 2014 | Quantitative study |
| Dawes[12] | 1994 | Quantitative study |
| Denis[13] | 2002 | Quantitative study |
| Falagas[14] | 2009 | Quantitative study |
| Farboud[15] | 2009 | Quantitative study |
| Fernandez[16] | 2010 | Quantitative study |
| Fowler[17] | 2012 | Quantitative study |
| Gett[18] | 2014 | Quantitative study |
| Ghane[19] | 2014 | Quantitative study |
| Ghulam[20] | 2006 | Quantitative study |
| Godinho[21] | 2010 | Opinion paper |
| Graham[22] | 1999 | No qualitative analysis |
| Habib[23] | 2008 | Quantitative study |
| Howlader[24] | 2004 | Quantitative study |
| Ihrig[25] | 2012 | Quantitative study |
| Jahan[26] | 2014 | Quantitative study |
| Jamjoom[27] | 2010 | Quantitative study |
| Janssen[28] | 2009 | Quantitative study |
| Kent[29] | 1996 | Quantitative study |
| King-Marshall[30] | 2016 | Does not deal with the consent process |
| Kiss[31] | 2004 | Quantitative study |
| Kortram[32] | 2016 | Protocol for prospective cohort study |
| Krupat[33] | 2000 | Quantitative study |
| Kusec[34] | 2006 | Does not deal with the consent process |
| Lescale[35] | 1996 | Quantitative study |
| Limbruno[36] | 2008 | Quantitative study |
| Loughran[37] | 2015 | Quantitative study |
| Mazur[38] | 1994 | Quantitative study |
| Mazur[39] | 1997 | Quantitative study |
| McCormack[40] | 1997 | Quantitative study |
| McFarlin[41] | 2004 | Literature review |
| Narumi[42] | 1998 | Quantitative study |
| O'Brien[43] | 2006 | Quantitative study |
| O'Neill[44] | 2007 | Does not deal with the consent process |
| Oosthuizen[45] | 2012 | Quantitative study |
| Park[46] | 2016 | Quantitative presentation of court proceedings |
| Radosa[47] | 2016 | Quantitative study |
| Sarela[48] | 2014 | Literature review |
| Saw[49] | 1994 | Quantitative study |
| Scanlan[50] | 2003 | Quantitative study |
| Sepucha[51] | 2010 | Quantitative study |
| Shannon[52] | 2008 | Quantitative study |
| Srinivasan[53] | 1999 | Quantitative study |
| Stanisic[54] | 2014 | No qualitative analysis |
| Tan[55] | 2008 | Quantitative study |
| Thurlow[56] | 1989 | Opinion paper |
| Wallace[57] | 1986 | Opinion paper |
| Weckbach[58] | 2016 | Quantitative study |
| Weinstein[59] | 2007 | Literature review |
| Wolf[60] | 2005 | Quantitative study |
| Wu[61] | 1988 | Quantitative study |

## References:

1. Ashraf B, Tasnim N, Saaiq M, Khaleeq-Uz-Zaman. Informed consent for surgery: do our current practices conform to the accepted standards?. J Coll Physicians Surg Pak. 2014;24:775–7.

2. H.B. B, K.M. S, S.C. G, Bosworth HB, Stechuchak KM, Grambow SC, et al. Patient risk perceptions for carotid endarterectomy: Which patients are strongly averse to surgery? J Vasc Surg. 2004;40:86–91.

3. Bowden MT, Church CA, Chiu AG, Vaughan WC. Informed consent in functional endoscopic sinus surgery: the patient’s perspective. Otolaryngol Head Neck Surg. 2004;131:126–32.

4. Braddock C 3rd, Hudak PL, Feldman JJ, Bereknyei S, Frankel RM, Levinson W. “Surgery is certainly one good option”: quality and time-efficiency of informed decision-making in surgery. J Bone Joint Surg Am. 2008;90:1830–8.

5. Brosnam T, Perry M. “Informed” consent in adult patients: can we achieve a gold standard?. Br J Oral Maxillofac Surg. 2009;47:186–90.

6. Byrne DJ, Napier A, Cuschieri A. How informed is signed consent?. Br Med J (Clin Res Ed). 1988;296:839–40.

7. Cawich SO, Barnett AT, Crandon IW, Drew SD, Gordon-Strachan G. From the patient’s perspective: is there a need to improve the quality of informed consent for surgery in training hospitals?. Perm J. 2013;17:22–6.

8. Clarke S, Oakley J. Informed consent and surgeons’ performance. Inf Consent Clin Account ethics Rep cards Surg performance. 2007;:111–33.

9. DM C, Dexter F, JJ P, TL T, EW N, SJ B. Patients’ perspective on full disclosure and informed consent regarding postoperative visual loss associated with spinal surgery in the prone position. Mayo Clin Proc. 2011;86:865–8.

10. Courtney MJ. Information about surgery: what does the public want to know?. ANZ J Surg. 2001;71:24–6.

11. S. D, L. G, J. A, J. R, S. S, E. F, et al. Informed consent for cardiac procedures: deficiencies in patient comprehension with current methods. Ann Thorac Surg. 2014;97:1502–5.

12. Dawes PJ, Davison P. Informed consent: what do patients want to know?. J R Soc Med. 1994;87:149–52.

13. Denis B, Bottlaender J, Goineau J, Peter A, Weiss A-M. [Informed consent for gastrointestinal endoscopy. A patient-opinion survey]. Consent eclaire en Endosc Dig Enq d’opinion aupres des Mal. 2002;26:675–9.

14. Falagas ME, Akrivos PD, Alexiou VG, Saridakis V, Moutos T, Peppas G, et al. Patients’ perception of quality of pre-operative informed consent in athens, Greece: a pilot study. PLoS One. 2009;4:e8073.

15. Farboud A, Ching H, Tomkinson A. Risk perception in consent for surgical procedures: a comparative analysis. J One-Day Surg. 2009;19:104–8.

16. Fernandez A. Improving the Quality of Informed Consent: It Is Not All About the Risks. Ann Intern Med. 2010;153:342–3.

17. Fowler FJJ, Gallagher PM, Bynum JPW, Barry MJ, Lucas FL, Skinner JS, et al. Decision-making process reported by medicare patients who had coronary artery stenting or surgery for prostate cancer. J Gen Intern Med. 2012;27:911–6.

18. Gett RM, Cooray AR, Gold D, Danta M, R.M. G, A.R. C, et al. Evaluating informed consent for colonoscopy. Surg Laparosc Endosc Percutaneous Tech. 2014;24:345–52.

19. Ghane A, Huynh HP, Andrews SE, Legg AM, Tabuenca A, Sweeny K. The relative importance of patients’ decisional control preferences and experiences. Psychol Health. 2014;29:1105–18.

20. AT G, Kessler M, LM B, Haller U, TM K, Ghulam AT, et al. Patients’ satisfaction with the preoperative informed consent procedure: a multicenter questionnaire survey in Switzerland. Mayo Clin Proc. 2006;81:307–12.

21. Godinho AM, Lanziotti LH, de Morais BS. Informed Consent: The Understanding of Lawyers and Courts. Rev Bras Anestesiol. 2010;60:207–14.

22. Graham WJ, Hundley V, McCheyne AL, Hall MH, Gurney E, Milne J. An investigation of women’s involvement in the decision to deliver by caesarean section. Br J Obstet Gynaecol. 1999;106:213–20.

23. SB H, Sonoda L, TC S, PJ E, AM G, Habib SB, et al. How do patients perceive the benefits and risks of peripheral angioplasty? Implications for informed consent. J Vasc Interv Radiol. 2008;19:177–81.

24. Howlader MH, Dhanji A-R, Uppal R, Magee P, Wood AJ, Anyanwu AC, et al. Patients’ views of the consent process for adult cardiac surgery: questionnaire survey. Scand Cardiovasc J. 2004;38:363–8.

25. Ihrig A, Herzog W, Huber CG, Hadaschik B, Pahernik S, Hohenfellner M, et al. Multimedia support in preoperative patient education for radical prostatectomy: The physicians’ point of view. Patient Educ Couns. 2012;87:239–42.

26. Jahan F, Roshan R, Nanji K, Sajwani U, Warsani S, Jaffer S. Factors affecting the process of obtaining informed consent to surgery among patients and relatives in a developing country: results from Pakistan. East Mediterr Health J. 2014;20:569–77.

27. Jamjoom AAB, White S, Walton SM, Hardman JG, Moppett IK, A.A. J, et al. Anaesthetists’ and surgeons’ attitudes towards informed consent in the UK: an observational study. BMC Med Ethics. 2010;11:2.

28. Janssen NBAT, Oort FJ, Fockens P, Willems DL, de Haes HCJM, Smets EMA. Under what conditions do patients want to be informed about their risk of a complication? A vignette study. J Med Ethics. 2009;35:276–82.

29. Kent G. Difficulties in obtaining informed consent by psychiatrists, surgeons and obstetricians/gynaecologists. Health Care Anal. 1996;4:65–71.

30. King-Marshall EC, Mueller N, Dailey A, Barnett TE, George TJJ, Sultan S, et al. “It is just another test they want to do”: Patient and caregiver understanding of the colonoscopy procedure. Patient Educ Couns. 2016;99:651–8.

31. C.G. K, S. R-M, E. S, G. D-R, M. V-P, Kiss CG, et al. Informed Consent and Decision Making by Cataract Patients. Arch Ophthalmol. 2004;122:94–8.

32. Kortram K, Ijzermans JNM, Dor FJMF. Towards a standardized informed consent procedure for live donor nephrectomy: What do surgeons tell their donors? Int J Surg. 2016;32:83–8.

33. Krupat E, Fancey M, Cleary PD. Information and its impact on satisfaction among surgical patients. Soc Sci Med. 2000;51:1817–25.

34. S. K, S. O, M. S, D. K, Z. B, Kusec S, et al. Improving comprehension of informed consent. Patient Educ Couns. 2006;60:294–300.

35. KB L, SR I, KA E, EQ P, FA C, LB M, et al. Conflicts between physicians and patients in non-elective cesarean delivery: incidence and the adequacy of informed consent. Am J Perinatol. 1996;13:171–6.

36. U L, AG E, F C, E R, C P, R M, et al. Usefulness of an audiovisual support to informed consent before percutaneous coronary intervention procedures. 2008. p. 726–32.

37. Loughran D. Surgical consent: the world’s largest Chinese Whisper? A review of current surgical consent practices. J Med Ethics. 2015;41:206–10.

38. DJ M, JF M, Mazur DJ, Merz JF. Patients’ interpretations of verbal expressions of probability: implications for securing informed consent to medical interventions. In: Behavioral sciences & the law. 1994. p. 417–26.

39. Mazur DJ, Hickam DH. Patients’ preferences for risk disclosure and role in decision making for invasive medical procedures. J Gen Intern Med. 1997;12:114–7.

40. McCormack D, Evoy D, Mulcahy D, Walsh M. An evaluation of patients comprehension of orthopaedic terminology: implications for informed consent. J R Coll Surg Edinb. 1997;42:33–5.

41. BL M, McFarlin BL. Elective cesarean birth: issues and ethics of an informed decision. J Midwifery Womens Health. 2004;49:421–9.

42. Narumi J, Miyazawa S, Miyata H, Suzuki A, Sugiura T, Oshiumi M, et al. Patients’ understanding and opinion about informed consent for coronary angiography in a rural Japanese hospital. Intern Med. 1998;37:18–20.

43. O’Brien CM, Thorburn TG, Sibbel-Linz A, McGregor AD. Consent for plastic surgical procedures. J Plast Reconstr Aesthet Surg. 2006;59:983–9.

44. O’Neill T, Jinks C, Ong BN. Decision-making regarding total knee replacement surgery: a qualitative meta-synthesis. BMC Health Serv Res. 2007;7:52.

45. Oosthuizen JC, Burns P, Timon C. The changing face of informed surgical consent. J Laryngol Otol. 2012;126:236–9.

46. Park BY, Kwon J, Kang SR, Hong SE. Informed Consent as a Litigation Strategy in the Field of Aesthetic Surgery: An Analysis Based on Court Precedents. Arch Plast SURGERY-APS. 2016;43:402–10.

47. Radosa JC, Radosa CG, Kastl C, Mavrova R, Gabriel L, Graeber S, et al. Influence of the Preoperative Decision-Making Process on the Postoperative Outcome after Hysterectomy for Benign Uterine Pathologies. Geburtshilfe Frauenheilkd. 2016;76:383–9.

48. Sarela A. Informed Decision-Making for Bariatric Surgery: Benefits, Risks, Uncertainties and Choices. Indian J Surg. 2014;76:467–73.

49. Saw KC, Wood AM, Murphy K, Parry JR, Hartfall WG. Informed consent: an evaluation of patients’ understanding and opinion (with respect to the operation of transurethral resection of prostate). J R Soc Med. 1994;87:143–4.

50. Scanlan D, Siddiqui F, Perry G, Hutnik CML, D. S, F. S, et al. Informed consent for cataract surgery: what patients do and do not understand. J Cataract Refract Surg. 2003;29:1904–12.

51. Sepucha KR, Fagerlin A, Couper MP, Levin CA, Singer E, Zikmund-Fisher BJ. How does feeling informed relate to being informed? The DECISIONS survey. Med Decis Making. 2010;30 5 Suppl:77S–84S.

52. Shannon H, Scott T. Patients’ perceptions of informed consent for surgical procedures in Northern Ireland: a retrospective survey. Br J Anaesth Recover Nurs. 2008;9:55–65.

53. Srinivasan J. Observing communication skills for informed consent: an examiner’s experience. Ann R Coll Physicians Surg Can. 1999;32:437–40.

54. M.-G. S. Reasons underlying the consent to endovascular treatment, displayed by patients diagnosed with asymptomatic internal carotid artery stenosis. Med Sci Monit. 2014;20:1503–9.

55. L.T. T, H. J, J. R-H. Should patients set the agenda for informed consent? A prospective survey of desire for information and discussion prior to routine cataract surgery. Therapeutics and Clinical Risk Management. 2008;4:1119–25.

56. JG T. Informed consent: every patient’s right. Gastroenterol Nurs. 1989;12:132–4.

57. Wallace LM. Informed consent to elective surgery: The “therapeutic” value? Soc Sci Med. 1986;22:29–33.

58. Weckbach S, Kocak T, Reichel H, Lattig F. A survey on patients’ knowledge and expectations during informed consent for spinal surgery: can we improve the shared decision-making process? PATIENT Saf Surg. 2016;10.

59. JN W, Clay K, TS M. Informed patient choice: patient-centered valuing of surgical risks and benefits. Health Aff. 2007;26:726–30.

60. Wolf JS, Chiu AG, Palmer JN, O’Malley BWJ, Schofield K, Taylor RJ. Informed consent in endoscopic sinus surgery: the patient perspective. Laryngoscope. 2005;115:492–4.

61. Wu WC, Pearlman RA. Consent in medical decision making: the role of communication. J Gen Intern Med. 1988;3:9–14.
