# Supplementary material for: The patient and clinician experience of informed consent for surgery: a systematic review of the qualitative evidence
Source: BMC Med Ethics. 2020 Jul 11;21:58. doi: 10.1186/s12910-020-00501-6 (PMC7353438; doi:10.1186/s12910-020-00501-6)
Supplement: Supplementary file 3 — Additional file 3. [file 12910_2020_501_MOESM3_ESM.docx]

| Citation | Q1 | Q2 | Q3 | Q4 | Q5 | Q6 | Q7 | Q8 | Q9 | Q10 |
| --- | --- | --- | --- | --- | --- | --- | --- | --- | --- | --- |
| Berman. 2008. | Y | Y | Y | Y | Y | N | U | Y | Y | Y |
| Bramall. 2014. | Y | Y | Y | Y | Y | Y | Y | Y | Y | Y |
| Dixon-Woods. 2006. | Y | Y | Y | Y | Y | Y | U | Y | Y | Y |
| Habiba. 2004. | Y | Y | Y | Y | Y | N | Y | Y | Y | Y |
| Hall. 2012. | Y | Y | Y | Y | Y | N | Y | Y | Y | Y |
| Kumar. 2012. | Y | Y | Y | Y | Y | Y | Y | Y | Y | Y |
| McKneally. 2009. | Y | U | Y | Y | Y | N | Y | Y | Y | Y |
| McKneally. 2004. | Y | Y | Y | U | Y | N | Y | Y | Y | Y |
| McKneally. 2000. | Y | Y | Y | Y | Y | N | Y | Y | Y | Y |
| Meredith. 1996. | U | U | Y | Y | Y | N | N | U | U | Y |
| Moore. 2002. | Y | Y | Y | Y | Y | Y | Y | Y | Y | Y |
| Schaufel. 2009. | Y | Y | Y | Y | Y | N | N | Y | Y | Y |
| Spector. 2010. | Y | Y | Y | Y | Y | N | N | Y | U | Y |
| Suarez-Almazor. 2010. | Y | Y | Y | Y | Y | N | Y | Y | N | Y |
| Wood. 2014. | Y | Y | Y | Y | Y | N | Y | Y | Y | Y |
| McNair. 2016. | Y | Y | Y | Y | Y | Y | Y | Y | Y | Y |
| % | 93.75 | 87.5 | 100.0 | 93.75 | 100.0 | 31.25 | 68.75 | 93.75 | 81.25 | 100.0 |

| 1. Is there congruity between the stated philosophical perspective and the research methodology? |
| --- |
| 1. Is there congruity between the research methodology and the research question or objectives? |
| 1. Is there congruity between the research methodology and the methods used to collect data? |
| 1. Is there congruity between the research methodology and the representation and analysis of data? |
| 1. Is there congruity between the research methodology and the interpretation of results? |
| 1. Is there a statement locating the researcher culturally or theoretically? |
| 1. Is the influence of the researcher on the research, and vice- versa, addressed? |
| 1. Are participants, and their voices, adequately represented? |
| 1. Is the research ethical according to current criteria or, for recent studies, and is there evidence of ethical approval by an appropriate body? |
| 1. Do the conclusions drawn in the research report flow from the analysis, or interpretation, of the data? |
