# Supplementary figures and images for: The patient and clinician experience of informed consent for surgery: a systematic review of the qualitative evidence
Source: BMC Med Ethics. 2020 Jul 11;21:58. doi: 10.1186/s12910-020-00501-6 (PMC7353438; doi:10.1186/s12910-020-00501-6)

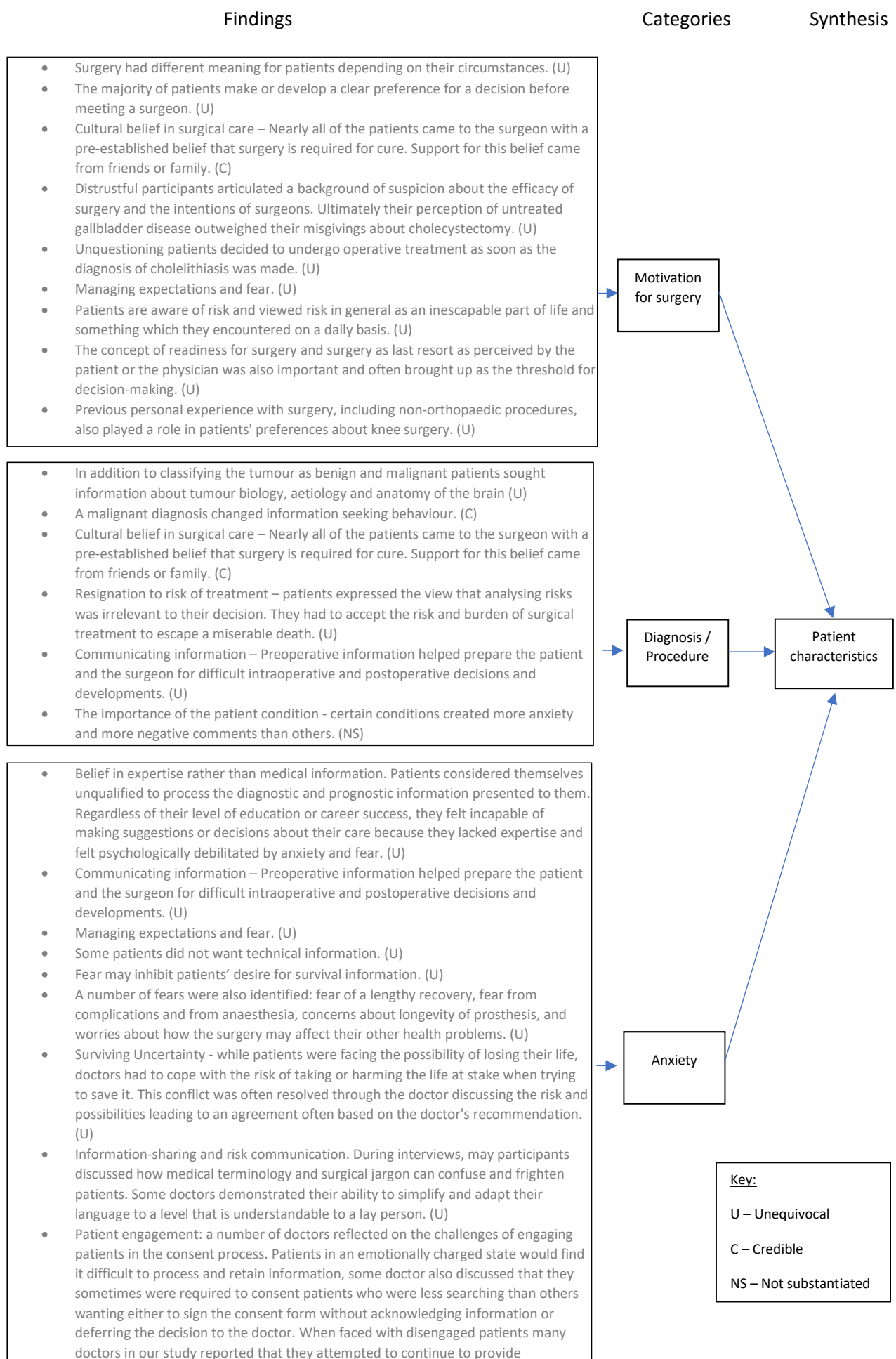

Findings

Categories

Synthesis

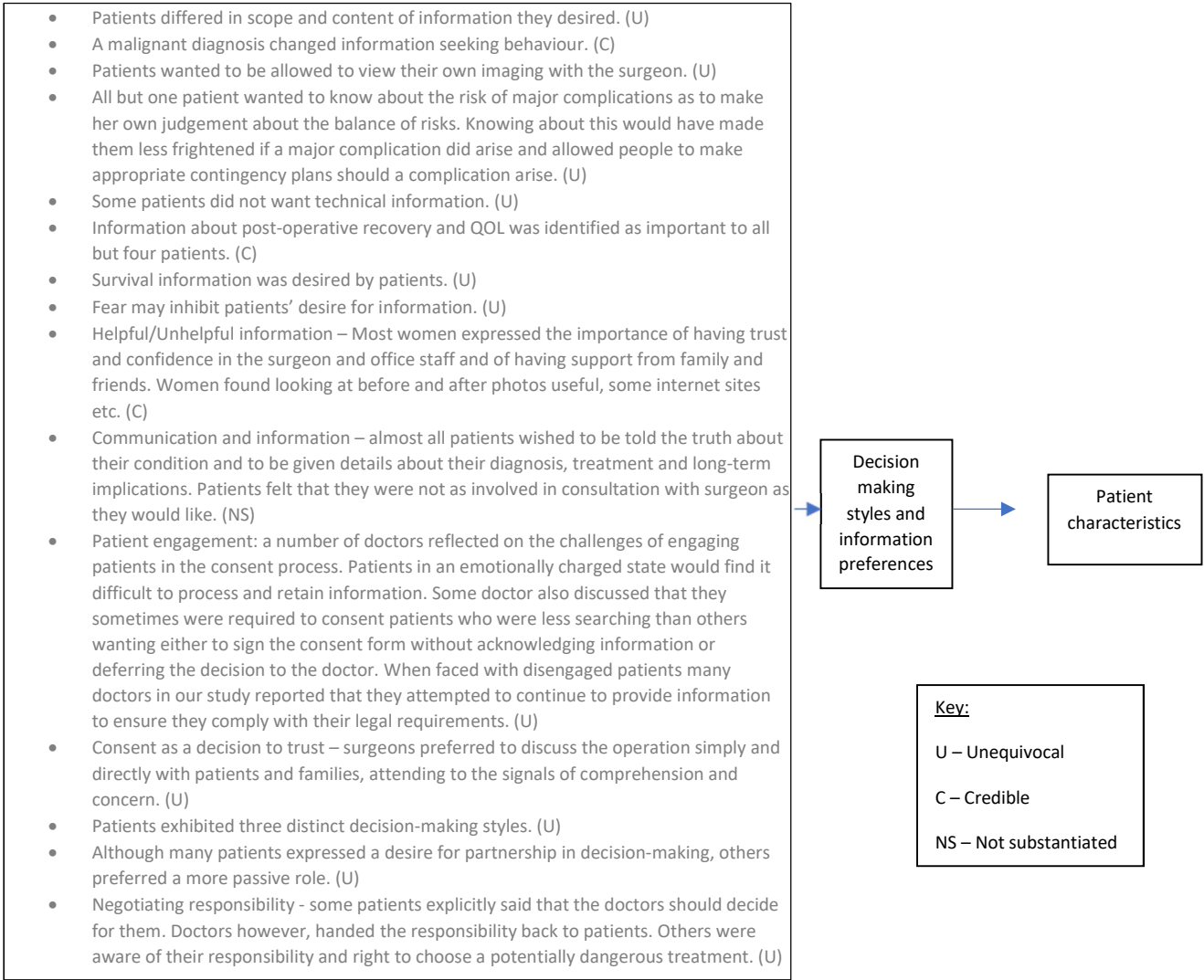

Figure 2: Patient characteristics synthesised finding.

Supplement: Supplementary file 4 — Additional file 4. [file 12910_2020_501_MOESM4_ESM.pdf]

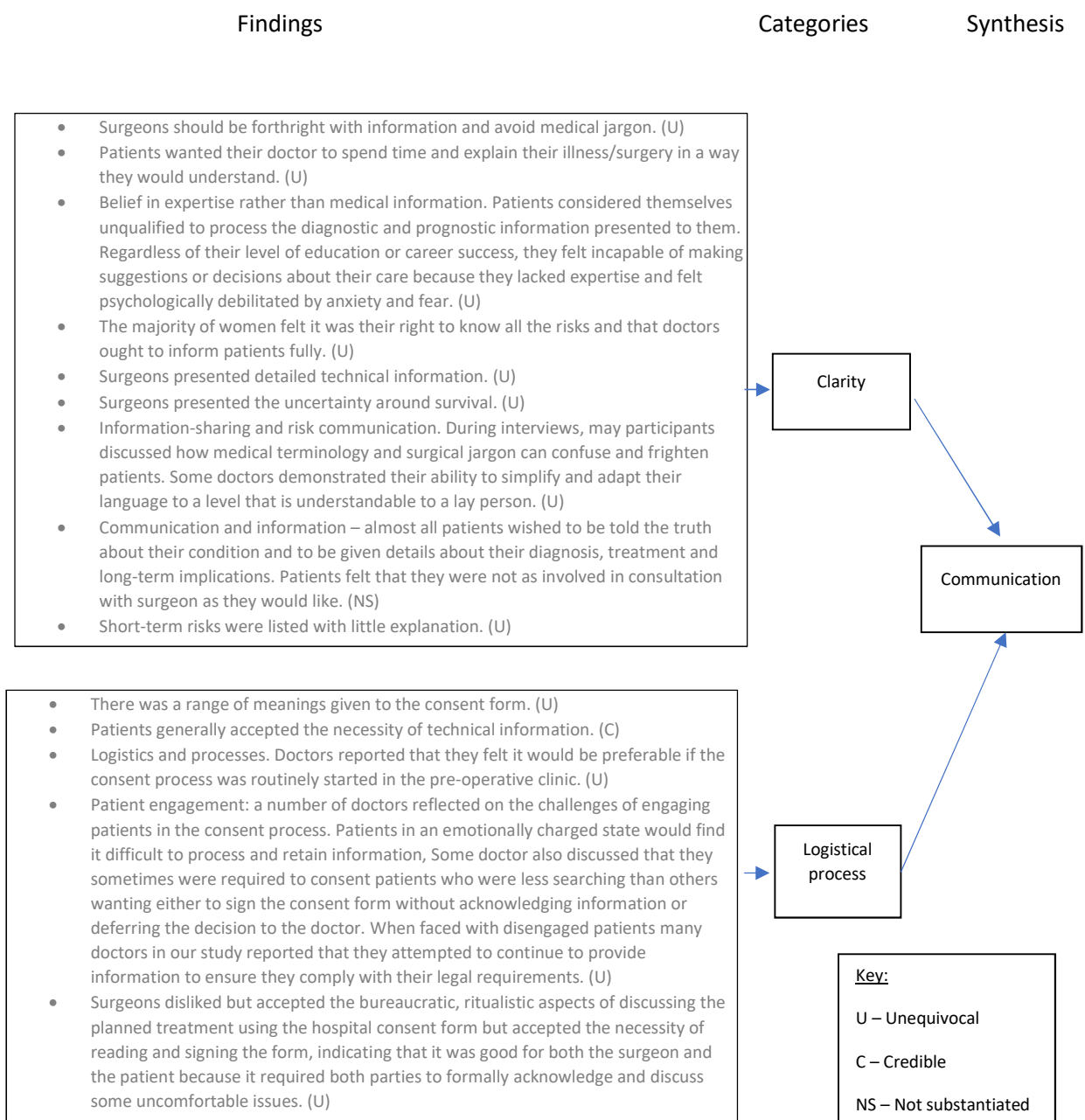

Figure 4: Communication synthesised finding.

Supplement: Supplementary file 6 — Additional file 6. [file 12910_2020_501_MOESM6_ESM.pdf]

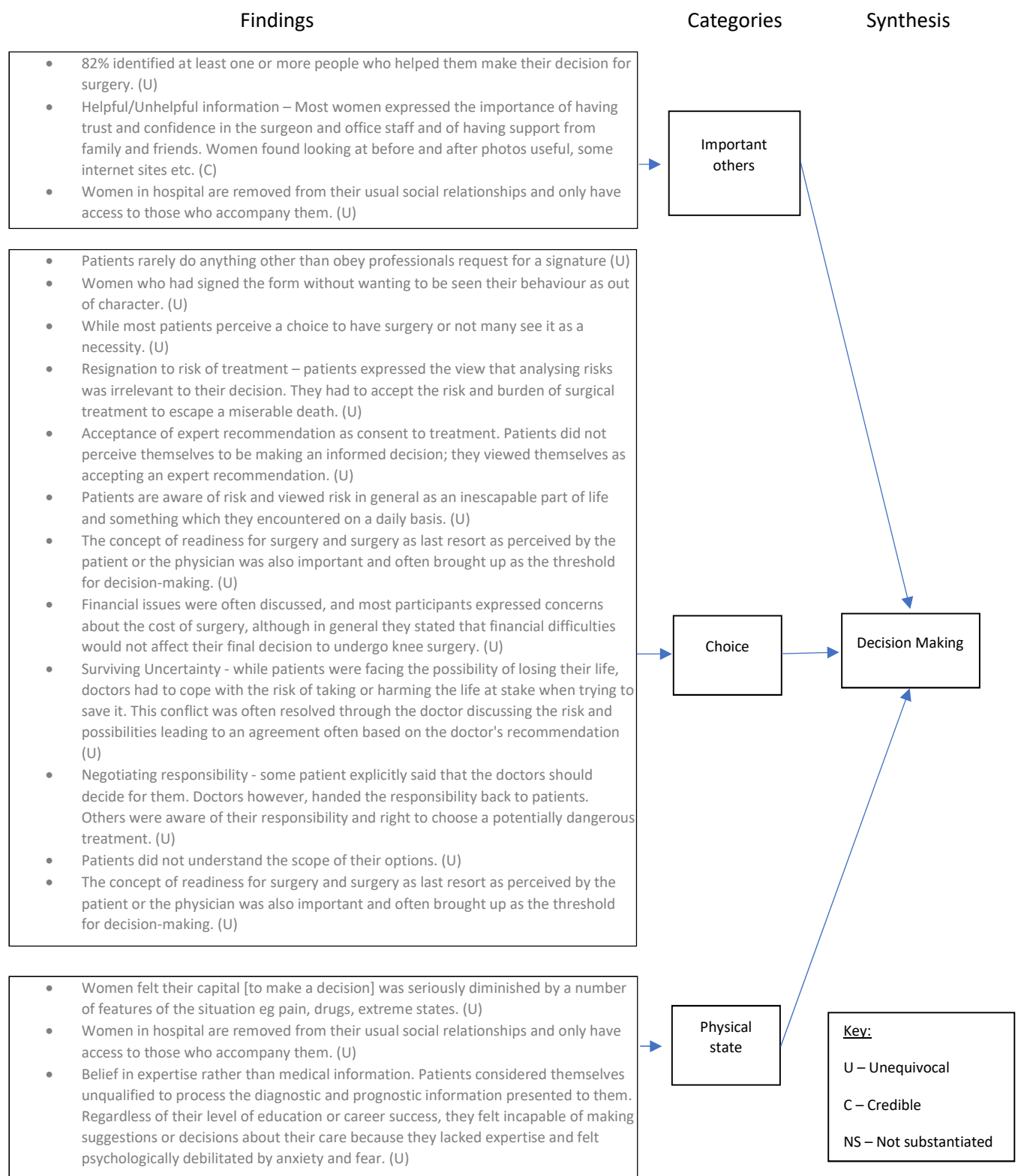

Figure 7: Decision making synthesised finding.

Supplement: Supplementary file 7 — Additional file 7. [file 12910_2020_501_MOESM7_ESM.pdf]
