# Supplementary material for: The patient and clinician experience of informed consent for surgery: a systematic review of the qualitative evidence
Source: BMC Med Ethics. 2020 Jul 11;21:58. doi: 10.1186/s12910-020-00501-6 (PMC7353438; doi:10.1186/s12910-020-00501-6)
Supplement: Supplementary file 5 — Additional file 5. [file 12910_2020_501_MOESM5_ESM.pdf]

Findings | Categories | Synthesis

- Patients were not adequately informed prior to making the decision. (U)
- Most patients felt doctors described the details of surgery in fair or deficient detail or softened the detail. (NS)
- Belief in expertise rather than medical information. Patients considered themselves unqualified to process the diagnostic and prognostic information presented to them. Regardless of their level of education or career success, they felt incapable of making suggestions or decisions about their care because they lacked expertise and felt psychologically debilitated by anxiety and fear. (U)
- Women volunteered that in the event of a complication their attitude tot litigation would have been influenced by whether or not they had been informed about the possibility beforehand. (C)
- Unexpected outcomes: most women described some aspect of the postsurgical experience as being unexpected and therefore they felt psychologically unprepared e.g. degree of pain, length of time with pain, itchy breasts, and numbness in or around the breasts. (C)
- Time provided and situation of outpatient meeting - Patients reported problems with the brevity of the outpatient meeting and expressed frustration over their unpreparedness to respond to what was revealed on this occasion. (U)
- A second opportunity to communicate- shortcomings with the consultation could have been mitigated by having the opportunity for a second consultation. (U)
- Consent as a decision to trust – surgeons preferred to discuss the operation simply and directly with patients and families, attending to the signals of comprehension and concern. (U)
- Unfamiliarity with procedures. Many junior doctors admitted to feeling inexperienced and ultimately lacking confidence to consent for procedure of which they had little or no exposure. (U)
- Long-term effects of surgery were minimized by surgeons (U)
- Short-term risks were listed with little explanation. (U)
- Patients wanted to be allowed to view their own imaging with the surgeon. (U)
- In addition to classifying the tumour as benign and malignant patients sought information about tumour biology, aetiology and anatomy of the brain. (U)

→ Feeling of being inadequately informed → Knowledge

- Patients were reluctant to ask questions of doctors for several reasons – faith in the doctor, appearing poorly in eyes of the doctor, or people believed it was the duty of the doctor to disclose all necessary information without the patient needing to ask. (C)
- Belief in expertise rather than medical information. Patients considered themselves unqualified to process the diagnostic and prognostic information presented to them. Regardless of their level of education or career success, they felt incapable of making suggestions or decisions about their care because they lacked expertise and felt psychologically debilitated by anxiety and fear. (U)
- Cautious patients were initially uncertain about the benefits of surgical intervention but did not dismiss it at the onset of deliberation. These participants deferred cholecystectomy to investigate fully the risks and benefits of all treatment option. They described their experience as a process of information gathering and reasoning, leading to progressive development of confidence. Cautious patients became well informed to the point that they did not view the consent interview as a source of information. (U)
- Making informed decisions – surgeons stated that operations require the patient and the surgeon to make an informed decision about undertaking the procedure. Surgeons outlined the necessary information to help the patient make a decision but recognised that frightened patients may be unable to make an objective decision. Important factors influencing a surgeon's decision to operate were hard findings e.g. pulmonary and cardiac function, tumour stage and soft findings. (U)

→ Essential level of disclosure → Knowledge

Key:

U – Unequivocal

C – Credible

NS – Not substantiated

Findings	Categories	Synthesis

Findings

- The majority of women felt it was their right to know all the risks and that doctors ought to inform patients fully. (U)
- Women felt the risk of death should be disclosed as it would be reassuring to know how low it is. (U)
- All but one patient wanted to know about the risk of major complications as to make her own judgement about the balance of risks. Knowing about this would have made them less frightened if a major complication did arise and allowed people to make appropriate contingency plans should a complication arise. (U)
- Women felt knowing the risk of having an inconclusive was important in the decision-making process. (U)
- Women volunteered that in the event of a complication their attitude to litigation would have been influenced by whether or not they had been informed about the possibility beforehand. (C)
- Patients generally accepted the necessity of technical information (C)
- Some patients did not want technical information(U)
- Survival information was desired by patients (U)
- Fear may inhibit patients' desire for survival information (U)
- Unexpected outcomes: most women described some aspect of the postsurgical experience as being unexpected and therefore they felt psychologically unprepared. E.g. degree of pain, length of time with pain, itchy breasts, and numbness in or around the breasts. (C)
- Helpful/Unhelpful information – Most women expressed the importance of having trust and confidence in the surgeon and office staff and of having support from family and friends. Women found looking at before and after photos useful, some internet sites etc. (C)
- Information-sharing and risk communication. During interviews, may participants discussed how medical terminology and surgical jargon can confuse and frighten patients. Some doctors demonstrated their ability to simplify and adapt their language to a level that is understandable to a lay person. (U)
- Surgeons disliked but accepted the bureaucratic, ritualistic aspects of discussing the planned treatment using the hospital consent form but accepted the necessity reading and signing the form, indicating that it was good for both the surgeon and the patient because it required both parties to formally acknowledge and discuss some uncomfortable issues (U)

→ **Essential level of disclosure** → **Knowledge**

- The internet was the most widely used resource for seeking medical information (C)
- Surgeons should provide a permanent record of the consultation and guide patients to reliable online information resources (U)
- Patients described gaining information about their decision from more than one source (U)
- Cautious patients were initially uncertain about the benefits of surgical intervention but did not dismiss it at the onset of deliberation. These participants deferred cholecystectomy to investigate fully the risks and benefits of all treatment options. They described their experience as a process of information gathering and reasoning, leading to progressive development of confidence. Cautious patients became well informed to the point that they did not view the consent interview as a source of information.(U)
- Patients obtained information about TKA from different sources which often included non-professional contacts, such as relatives or acquaintances, the media (e.g. Television), and the Internet. (U)
- Helpful/Unhelpful information – Most women expressed the importance of having trust and confidence in the surgeon and office staff and of having support from family and friends. Women found looking at before and after photos useful, some internet sit etc. (C)
- A second opportunity to communicate - shortcomings with the consultation could have been mitigated by having the opportunity for a second consultation. (U)
- The role of printed information in communication - patients expressed an interest in more written materials. (C)

→ **Additional sources of information** → **Knowledge**

<u>Key:</u>

U – Unequivocal

C – Credible

NS – Not substantiated

Figure 3: Knowledge synthesised finding.
